# Nonlinear Regression Modelling: A Primer with Applications and Caveats

**DOI:** 10.1007/s11538-024-01274-4

**Published:** 2024-03-15

**Authors:** Timothy E. O’Brien, Jack W. Silcox

**Affiliations:** 1 Department of Mathematics and Statistics, Loyola University Chicago, Chicago, IL USA; 2 Department of Psychology, University of Utah, Salt Lake City, UT USA

**Keywords:** Bioassay, Dose–response, Likelihood ratio test, Profile likelihood confidence interval, Relative potency, Wald statistic

## Abstract

Use of nonlinear statistical methods and models are ubiquitous in scientific research. However, these methods may not be fully understood, and as demonstrated here, commonly-reported parameter p-values and confidence intervals may be inaccurate. The gentle introduction to nonlinear regression modelling and comprehensive illustrations given here provides applied researchers with the needed overview and tools to appreciate the nuances and breadth of these important methods. Since these methods build upon topics covered in first and second courses in applied statistics and predictive modelling, the target audience includes practitioners and students alike. To guide practitioners, we summarize, illustrate, develop, and extend nonlinear modelling methods, and underscore caveats of Wald statistics using basic illustrations and give key reasons for preferring likelihood methods. Parameter profiling in multiparameter models and exact or near-exact versus approximate likelihood methods are discussed and curvature measures are connected with the failure of the Wald approximations regularly used in statistical software. The discussion in the main paper has been kept at an introductory level and it can be covered on a first reading; additional details given in the Appendices can be worked through upon further study. The associated online Supplementary Information also provides the data and R computer code which can be easily adapted to aid researchers to fit nonlinear models to their data.

**Supplementary Information:**

The online version contains supplementary material available at 10.1007/s11538-024-01274-4.

## Introduction

Facilitated by readily-available statistical software, practitioners in fields as diverse as agronomy, biochemistry, biomedicine, drug development, engineering, environmental science, neuroscience, pharmacology and toxicology fit nonlinear models to their data to help answer their research questions. In addition to providing parsimonious data fits, nonlinear models are preferred to empirical models since the associated nonlinear model parameters typically have meaningful practical interpretations. For example, two drugs or experimental conditions are often compared in terms of half maximal effective concentration (${EC}_{50}$) or median lethal doses (${LD}_{50}$), which can be modelled as nonlinear dose–response model parameters. Mechanistic nonlinear models are often chosen based on underlying subject-matter knowledge such as Michaelis–Menten enzyme kinetic theory or dose response modelling methods (Bates and Watts 2007; Finney 1978; Govindarajulu 2001; Hubert 1992; Miguez et al. 2020; Ratkowsky 1983; Seber and Wild 1989). Faced with a plethora of experimental design and modelling methods, even statistically-savvy subject-matter practitioners may be unaware of key nonlinear methods and important requirements and cautions associated with nonlinear regression hypothesis testing methodologies and confidence interval estimation techniques.

Using straightforward nonlinear regression models and illustrations, this article overviews and illustrates useful nonlinear regression methods, underscores problems associated with commonly-used Wald statistic test p-values and confidence intervals (Wald 1943), and demonstrates the preference for exact likelihood-based confidence intervals over Wald intervals. Specifically, as highlighted below, p-values provided by statistical software packages are often based on the Wald approximation and can be grossly inaccurate for small- to moderately-sized studies. For example, Wald-based confidence intervals for nonlinear model parameters with nominal labels of 95% may have actual coverage levels of 75% or even lower. Conversely, readily-available exact or near-exact likelihood-based intervals generally show good agreement between nominal and actual coverage levels.

This nonlinear modelling introductory discussion, which builds upon readers background in linear methods (Draper and Smith 1998; Kleinbaum et al. 2014; Mendenhall and Sincich 2020), also provides the basis for further consideration of additional topics including dose response modelling, high-throughput screening methods, compartmental models based on differential equation(s) and other multivariate nonlinear models, computational algorithms and starting values, and further explorations of curvature measures. Given the evolution away from hypothesis testing approaches to estimation methods (Halsey 2019; Krzywinski and Altman 2013; Meeker and Escobar 1995), the focus here is largely on accurate confidence interval methods instead of p-values.

The article is structured as follows. To provide important context, Sect. 2 introduces simple motivating nonlinear model examples which highlight both nonlinear modelling in practice and underscores key differences with linear models. Section 3 overviews general nonlinear regression methods, makes connections to and contrasts with linear models, discusses parameter profiling in multiparameter models, nonlinear model selection, model fitting algorithms, and starting value selection. Section 4 provides additional exemplary nonlinear illustrations and extensions. In Sect. 5, we give important concluding remarks and discussion. The Appendices provide additional details and illustrations regarding the effects of curvature on nonlinear modelling, the Fieller-Creasy ratio of means example, comparisons of the F-based and the asymptotic likelihood tests and intervals, and comments and caveats regarding overfitting. Further, the R computer code (R Core Team 2020) used in the data analyses is given in the Supplementary Information. These R programs are easily adapted to help practitioners fit meaningful nonlinear models to their data.

## Motivating Illustrations

The two key motivating examples introduced and discussed here illustrate the basic use of nonlinear modelling and demonstrate some of the widespread use of these methods.

### Example 1

**Fig. 1:**
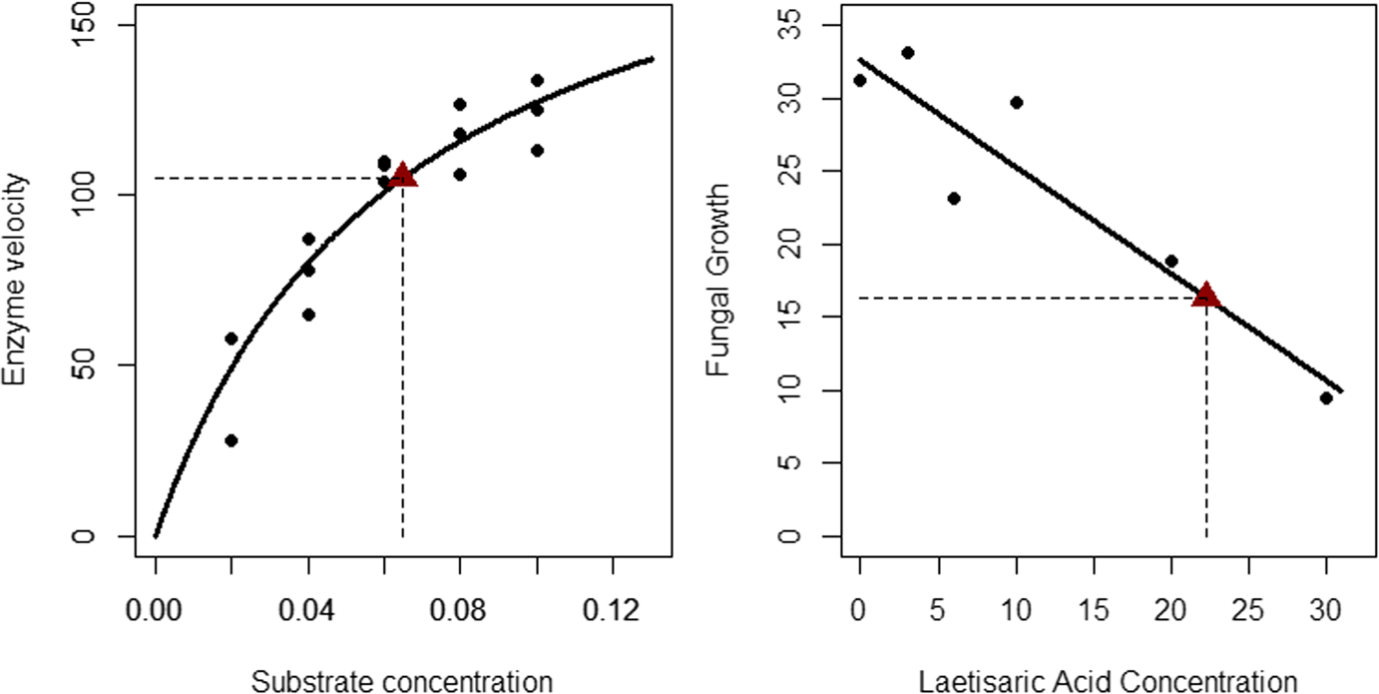
Two motivating example plots. Left panel: Plot of simulated data (filled circles), fitted two-parameter Michaelis–Menten model function and estimated ${EC}_{50}$ point (large, filled triangle). Right panel: Plot of fungal growth data (filled circles), fitted line and estimated ${IC}_{50}$ point (large, filled triangle)

For a single substrate, Michaelis–Menten enzyme kinetics theory (Michaelis and Menten 1913; Bates and Watts 2007) can be used to model the connection between the velocity of an enzymatic reaction (in counts per min^2^) to the substrate concentration (in ppm). To illustrate, consider the simulated data plotted in the left panel of Fig. 1. The chosen design involves the chosen substrate concentrations $x=0.02, 0.04, 0.06, 0.08$ and $0.10$ replicated three times. The reader can see that there is an obvious curve in the data and a researcher may be inclined to use a linear approach by modelling the data with a polynomial regression that includes a quadratic term in which the predictor is the squared substrate concentrations. This approach may help the model to successfully fit the data by accounting for the curvature seen but polynomial regression is notoriously difficult to interpret. Instead, if a researcher were to use a nonlinear approach that was motivated by theory, such as the Michaelis–Menten enzyme kinetic theory, they would have a much more useful and interpretable model.

The classical Michaelis–Menten model function is given by

1 $$\eta \left(x,{\varvec{\theta}}\right)=\frac{{\theta }_{1}x}{{\theta }_{2}+x}$$

Here, the model parameters are $${\varvec{\theta}}=\left(\begin{array}{c}{\theta }_{1}\\ {\theta }_{2}\end{array}\right)$$, where ${\theta }_{1}$ is the upper asymptote (also called the ultimate velocity parameter) and ${\theta }_{2}$ is the ${EC}_{50}$ or ${IC}_{50}$ (sometimes also called the half-velocity) parameter. This follows since when $x={\theta }_{2}$, we obtain $\eta \left(x,{\varvec{\theta}}\right)={\frac{1}{2}\theta }_{1}$. By connecting the substrate concentrations (${x}_{i}$) with the velocity reaction rate (${y}_{ij}$) data using the additive model expression ${y}_{ij}=\eta \left({x}_{i},{\varvec{\theta}}\right)+{\varepsilon }_{ij}$ and least-squares estimation (see Sect. 3 and the R-code in the Supplementary Information), we obtain the parameter estimates, ${\widehat{\theta }}_{1}=209.868$ and ${\widehat{\theta }}_{2}=0.0647$. This fitted Michaelis–Menten model function is the nonlinear curve plotted in the left panel of Fig. 1. This model predicts that the enzyme velocity levels off at an ultimate velocity (upper asymptote) of almost 210 counts per min^2^. It also predicts that for a substrate concentration of $0.0647$ ppm, the predicted velocity is approximately half of $210$ or about $105$ counts per min^2^. Note that the half-velocity point $(0.0647, 104.9)$ is the plotted solid triangle and is highlighted by the dashed vertical and horizontal lines. ■

The above simple illustration demonstrates the common application of nonlinear modelling in practical applications. A researcher now has easy-to-interpret parameter estimates that fit within a theoretical model that was motivated by expert background knowledge rather than some arbitrary quadratic term from a polynomial linear regression that is difficult to interpret. The next example shows that nonlinear modelling is also encountered when fitting a linear model but where interest centers on a nonlinear function of the linear model parameters.

### Example 2

The data plotted in the right panel of Fig. 1 are adapted from a regression study (Bowers et al. 1986; Samuels et al. 2016) relating laetisaric acid concentration (the independent variable) to fungal growth (the dependent variable) in *P. ultimum,* the plant pathogen. These $n=6$ data points are plotted along with the fitted ordinary least-squares regression line. Indeed, a simple linear regression could be used to describe the relationship between laetisaric acid concentration and fungal growth in P. ultimum. However, the main goal of this particular study, as stated by the authors, was to estimate the acid concentration which “inhibits growth of P. ultimum by 50 percent” (Bowers et al. 1986, p. 106). Nonlinear regression modeling can help the authors meet this goal much more precisely. Rather than fitting a linear regression model, ${y}_{i}=\alpha +\beta {x}_{i}+{\varepsilon }_{i}$, the researchers could fit a nonlinear model that directly estimates the half maximal inhibitory concentration (sometimes referred to as ${IC}_{50}$). In the following nonlinear model, this ${IC}_{50}$ parameter is represented by theta ($\theta$) and the expected zero-concentration value (i.e., the intercept) is represented by alpha ($\alpha$):

2 $$\eta \left(x,{\varvec{\theta}}\right)=\alpha \left(1-\frac{x}{2\theta }\right)$$

We can fit this model using the”nls” function in R (for more details, please see the code in the Supplementary Information). This model fit shows that the parameter estimate for alpha (i.e., the intercept) is$\widehat{\alpha }=32.64$. In other words, the expected fungal growth with no applied laetisaric acid is estimated to be 32.64. Our model fit also estimates that$\widehat{\theta }=22.33$. In other words, the concentration of laetisaric acid that will inhibit P. ultimum growth by half is 22.33. Indeed, if the reader plugs 22.33 into $x$ in our fitted model, the expected value of$y$, or${\widehat{y}}_{50}=\eta \left(\widehat{\theta }\right)=16.32$, is indeed half of $\widehat{\alpha }$ (i.e., the estimated intercept).

The observant reader may note that the fit for this model (depicted as a black line in the right panel of Fig. 1) is indeed a straight line. That is, if a person were to fit the data using the”lm” function in R, they would end up with essentially the same line as the one seen in Fig. 1. So, it is natural to ask why this model is considered nonlinear considering it produces a fit that is a straight line. As discussed further in Sect. 3, nonlinearity refers to whether or not the parameters of the model *enter the model linearly or nonlinearly*. In the Eq. (2), *note that the slope (parameter expression which multiplies*$x$*) is*$-\frac{\alpha }{2\theta }$*, which is nonlinear in the model parameters*. Therefore, the model introduced in Eq. (2) is nonlinear.

The reader may next ask what benefit they gain from fitting a nonlinear model in this scenario since the produced model fit is the same line that a linear model would produce. When fitting a linear model in this case, the parameters that are estimated are the intercept (which is also estimated in the Eq. (2) model) and the slope of the line. This slope estimate would tell the researcher how much growth would change, on average, for every one unit increase in laetisaric acid concentration. This may be of interest to the researcher. But, as discussed above, the main goal of the study, as stated by the authors, was to investigate at which concentration of acid they may expect to see a 50% decline in growth. This so-called ${IC}_{50}$ value (i.e., theta) *could* be determined from the linear model but the researcher would be unable to perform hypothesis testing or to calculate confidence intervals without making often inaccurate simplifying approximations. With the nonlinear model, we are *directly* estimating the ${IC}_{50}$ parameter and, therefore, may directly test hypotheses and estimate confidence intervals.

In Fig. 1’s right panel, the point $(\widehat{\theta },{\widehat{y}}_{50})$ (the filled triangle) as well as the corresponding vertical and horizontal (dashed) line segments are also plotted. As mentioned above, because we now have a direct estimate of the ${IC}_{50}$ from our model we now calculate a confidence interval for this estimate that is so important for our study. ■

In this paper, we will discuss two different approaches to creating confidence intervals: the traditional Wald approach and the likelihood-based approach. As noted in the next section, for linear models, the two approaches generally give the same estimates for confidence intervals. But, as we will detail in Sect. 3 and elsewhere in the paper, likelihood-based confidence intervals are typically preferable when using nonlinear models.

## Key Nonlinear Regression Methods and Results

In this section, we briefly introduce and develop key nonlinear regression results. Additional details, including theoretical results, are given in general nonlinear texts (Bates and Watts 2007; Ratkowsky 1983; Seber and Wild 1989) and in subject-matter works from an array of fields including agronomy (Miguez et al. 2020), animal science (Gonçalves et al. 2016), immunology (Bursa et al. 2020), and pupillometry (Bartošová et al. 2018; Rollins et al. 2014; You et al. 2021). Before addressing nonlinear models, we first illustrate various linear models.

For $i=\mathrm{1,2},\dots n$, the usual (homoscedastic normal) simple linear regression model is written ${y}_{i}=\alpha +\beta {x}_{i}+{\varepsilon }_{i}$ with ${\varepsilon }_{i}{ \sim }_{iid} N\left(0,{\sigma }^{2}\right)$ where “$iid$” denotes ‘independent and identically distributed’. For the model function $\eta \left({x}_{i},{\varvec{\theta}}\right)=\alpha +\beta {x}_{i}$ and model function parameter vector $${\varvec{\theta}}=\left(\begin{array}{c}\alpha \\ \beta \end{array}\right)$$, the general structure of this model is

3 $${y}_{i}=\eta \left({x}_{i},{\varvec{\theta}}\right)+{\varepsilon }_{i}$$

The multiple linear regression model function is $\eta \left({x}_{i},{\varvec{\theta}}\right)=\alpha +{\beta }_{1}{x}_{i1}+{\beta }_{2}{x}_{i2}+\dots +{\beta }_{p-1}{x}_{i\left(p-1\right)}$ for ${{\varvec{\theta}}}^{T}=\left(\alpha ,{\beta }_{1},\dots ,{\beta }_{p-1}\right)$ and the quadratic regression model is $\eta \left({x}_{i},{\varvec{\theta}}\right)=\alpha +{\beta }_{1}{x}_{i}+{\beta }_{2}{x}_{i}^{2}$.

Perhaps surprisingly, even though the quadratic regression model (and other polynomial models) is sometimes used to account for curves observed in data, it is actually a *linear* model. In Sect. 2, we also saw a case in which we used a *nonlinear* model to fit a straight line to data with no observable curvature in it (see Example 2). So, what exactly do we mean when we call a model “nonlinear” since it does not necessarily refer to the shape that we see in the data? We next define and illustrate nonlinearity in regression modelling.

### What Makes a Nonlinear Model Function Nonlinear?

For the homoscedastic normal model given in Eq. (3), the model function $\eta \left({x}_{i},{\varvec{\theta}}\right)$ with parameters ${{\varvec{\theta}}}^{T}=\left({\theta }_{1},{\theta }_{2},\dots ,{\theta }_{p}\right)$ is characterized as nonlinear if the (partial) derivative of $\eta \left({x}_{i},{\varvec{\theta}}\right)$ with respect to at least one of the parameters includes at least one of the model parameters. For example, for the Michaelis–Menten model function from Example 1 in Sect. 2, $\eta \left(x,{\varvec{\theta}}\right)=\frac{{\theta }_{1}x}{{\theta }_{2}+x}$, note that both the partial derivatives, $\frac{\partial \eta }{\partial {\theta }_{1}}=\frac{x}{{\theta }_{2}+x}$ and $\frac{\partial \eta }{\partial {\theta }_{2}}=-\frac{{\theta }_{1}x}{{\left({\theta }_{2}+x\right)}^{2}}$, contain model parameters, so this model function is nonlinear. Similarly, for Example 2 with model function $\eta \left(x,{\varvec{\theta}}\right)=\alpha \left(1-\frac{x}{2\theta }\right)$, this model function is nonlinear since at least one of the two derivatives, $\frac{\partial \eta }{\partial \alpha }=\left(1-\frac{x}{2\theta }\right)$ and $\frac{\partial \eta }{\partial \theta }=\frac{\alpha x}{2{\theta }^{2}}$, contain model parameters—in this case, both partial derivatives do. On the other hand, the quadratic model function $\eta \left(x,{\varvec{\theta}}\right)=\alpha +{\beta }_{1}x+{\beta }_{2}{x}^{2}$ has derivatives $\frac{\partial \eta }{\partial \alpha }=1, \frac{\partial \eta }{\partial {\beta }_{1}}=x$ and $\frac{\partial \eta }{\partial {\beta }_{2}}={x}^{2}$, and since none of these contain model parameters, the quadratic model is indeed a *linear* model. In sum, nonlinearity assesses the manner in which the model function parameters enter the model function—not how the explanatory variable(s) enter.

Another way in which nonlinear models are encountered is when a linear model is fit but where interest focuses on a nonlinear function of the model parameters. This was the case for Example 2 where researchers fit the simple linear model function $\eta \left(x,{\varvec{\theta}}\right)=\alpha +\beta x$ but where their focus was on estimating the ${IC}_{50}$ parameter ($\theta$). This parameter is such that$\frac{\alpha }{2}=\alpha +\beta \theta$. Solving for$\beta$, we get$\beta =-\frac{\alpha }{2\theta }$, and when this value is substituted into $\eta \left(x,{\varvec{\theta}}\right)=\alpha +\beta x$ with $${\varvec{\theta}} = \left( {\begin{array}{*{20}c} \alpha \\ \beta \\ \end{array} } \right)\,,{\text{ this yields}}\,\eta \left( {x, {\varvec{\theta}}} \right) = \alpha \left( {1 - \frac{x}{2\theta }} \right)\,{\text{with}}\,{\varvec{\theta}} = \left( {\begin{array}{*{20}c} \alpha \\ \theta \\ \end{array} } \right).$$

Another example is when researchers fit the quadratic regression model and wish to estimate the input value $x$ where the model function achieves its maximum or minimum. Using basic calculus, this value, denoted $\delta$, is such that ${\beta }_{1}+2{\beta }_{2}\delta =0$, so that ${\beta }_{1}=-2{\beta }_{2}\delta$. The model function can then be rewritten $\eta \left(x,{\varvec{\theta}}\right)=\alpha -2{\beta }_{2}\delta x+{\beta }_{2}{x}^{2}$. This new way of writing the original quadratic model function, called a model reparameterization, yields a nonlinear model. It has the clear advantage of making the parameter of interest be an inherent model parameter so as to more easily obtain accurate point and interval estimates.

### Parameter Estimation: Point Estimates and Standard Errors

Parameter estimation for the homoscedastic normal nonlinear models considered here can be achieved using maximum likelihood estimation, or equivalently, least squares estimation. For $S\left({\varvec{\theta}}\right)$ given below in Eq. (5), the corresponding log-likelihood is written,

4 $$LL\left({\varvec{\theta}}\right)=-\frac{n}{2}{\text{log}}\left({\sigma }^{2}\right)-\frac{1}{2{\sigma }^{2}}S\left({\varvec{\theta}}\right)$$

Since the model function parameters only appear in the $S\left({\varvec{\theta}}\right)$ term, maximum likelihood estimates (denoted MLEs) can be found by minimizing the sum of squares function,

5 $$S\left({\varvec{\theta}}\right)={\sum }_{i=1}^{n}{\varepsilon }_{i}^{2}={\sum }_{i=1}^{n}{\left({y}_{i}-\eta \left({x}_{i},{\varvec{\theta}}\right)\right)}^{2}$$

Least-squares estimates (denoted LSEs) are those parameter values that minimize $S\left({\varvec{\theta}}\right)$ for each of the $p$ model function parameters. In other words, the goal of least squares estimation is to find parameter estimates that minimize the difference between observed values of *y* (denoted as ${y}_{i}$) and model-predicted values of *y* (typically denoted as ${\widehat{y}}_{i}$). We denote the MLE/LSE parameter vector by $\widehat{{\varvec{\theta}}}$, and, when transposed, we can write ${\widehat{{\varvec{\theta}}}}^{T}=\left({\widehat{\theta }}_{1},{\widehat{\theta }}_{2},\dots ,{\widehat{\theta }}_{p}\right)$. So for nonlinear model parameters, MLEs and LSEs are indeed the same.

Under standard regularity conditions, least-squares parameter estimates for these model function parameters are obtained by differentiating $S\left({\varvec{\theta}}\right)$ with respect to the $p$ model parameters, setting these derivatives to zero, and solving the resulting $p$ so-called normal equations. These normal equations are $\frac{\partial S({\varvec{\theta}})}{\partial {\theta }_{1}}=0,\frac{\partial S({\varvec{\theta}})}{\partial {\theta }_{2}}=0,\dots ,\frac{\partial S({\varvec{\theta}})}{\partial {\theta }_{p}}=0$, and they can also be written,

6 $${\sum }_{i=1}^{n}\frac{\partial \eta \left({x}_{i},\widehat{{\varvec{\theta}}}\right)}{\partial {\theta }_{1}}{e}_{i}=0,{\sum }_{i=1}^{n}\frac{\partial \eta \left({x}_{i},\widehat{{\varvec{\theta}}}\right)}{\partial {\theta }_{2}}{e}_{i}=0,\dots , {\sum }_{i=1}^{n}\frac{\partial \eta \left({x}_{i},\widehat{{\varvec{\theta}}}\right)}{\partial {\theta }_{p}}{e}_{i}=0,$$

where for $i=\mathrm{1,2},\dots ,n$, the model residuals are ${e}_{i}={y}_{i}-\eta \left({x}_{i},\widehat{{\varvec{\theta}}}\right)$. Note that in general for nonlinear model functions, Eq. (6) is a nonlinear system of $p$ equations in $p$ unknowns (i.e., the model function parameters). Since the system of normal equations can be written more concisely in matrix form, we introduce the $n\times p$ so-called Jacobian matrix,

7 $${\varvec{X}}=\left[\begin{array}{ccc}\frac{\partial \eta \left({x}_{1},\widehat{{\varvec{\theta}}}\right)}{\partial {\theta }_{1}}& \frac{\partial \eta \left({x}_{1},\widehat{{\varvec{\theta}}}\right)}{\partial {\theta }_{2}}& \begin{array}{cc}\cdots & \frac{\partial \eta \left({x}_{1},\widehat{{\varvec{\theta}}}\right)}{\partial {\theta }_{p}}\end{array}\\ \frac{\partial \eta \left({x}_{2},\widehat{{\varvec{\theta}}}\right)}{\partial {\theta }_{1}}& \frac{\partial \eta \left({x}_{2},\widehat{{\varvec{\theta}}}\right)}{\partial {\theta }_{2}}& \begin{array}{cc}\cdots & \frac{\partial \eta \left({x}_{2},\widehat{{\varvec{\theta}}}\right)}{\partial {\theta }_{p}}\end{array}\\ \begin{array}{c}\vdots \\ \frac{\partial \eta \left({x}_{n},\widehat{{\varvec{\theta}}}\right)}{\partial {\theta }_{1}}\end{array}& \begin{array}{c}\vdots \\ \frac{\partial \eta \left({x}_{n},\widehat{{\varvec{\theta}}}\right)}{\partial {\theta }_{2}}\end{array}& \begin{array}{cc}\ddots & \vdots \\ \cdots & \frac{\partial \eta \left({x}_{n},\widehat{{\varvec{\theta}}}\right)}{\partial {\theta }_{p}}\end{array}\end{array}\right]$$

Using this notation, the normal equations system of $p$ equations from Eq. (6) can be rewritten,

8 $${{\varvec{X}}}^{T}{\varvec{e}}=0,$$

where ${{\varvec{e}}}^{T}=\left({e}_{1},{e}_{2},\dots ,{e}_{n}\right)$. Readers familiar with linear models (where the model parameter vector is often denoted ${\varvec{\beta}}$) will recognize that here, ${\varvec{e}}={\varvec{y}}-{\varvec{X}}\widehat{{\varvec{\beta}}}$ and the normal equations are ${{\varvec{X}}}^{T}\left({\varvec{y}}-{\varvec{X}}\widehat{{\varvec{\beta}}}\right)=0$ or ${{\varvec{X}}}^{T}{\varvec{X}}\widehat{{\varvec{\beta}}}={{\varvec{X}}}^{T}{\varvec{y}}$. We emphasize that this latter expression holds only for linear models whereas Eq. (8) holds for the more general nonlinear model situation.

For the nonlinear models considered here, parameter estimates are obtained by solving the (nonlinear) normal equation system in Eq. (8) and this in general involves using numerical methods and computer algorithms such as the root-finding methods available in freeware packages such as R (see the Supplementary Information). The following two illustrations are provided to demonstrate the use of these nonlinear normal equations in nonlinear parameter estimation.

#### Example 3

Similar to the Michaelis-Menton model function in Example 1, consider the one-parameter (i.e., $p=1$) model function (which is nonlinear since the derivative contains the parameter $\theta$),

9 $$\eta \left(x,\theta \right)=\frac{x}{\theta +x}$$

Notice that this model function has $y=1$ as its upper asymptote and the model parameter $\theta$ is the model ${EC}_{50}$ since for $x=\theta , \eta \left(x,\theta \right)=\frac{1}{2}$. For this single parameter nonlinear illustration, we use the simulated $n=4$ data points, $\left({x}_{i},{y}_{i}\right)=\left(0, 0.037\right), \left(2, 0.209\right), (4, 0.519)$ and $(6, 0.430)$. Since $\frac{\partial \eta }{\partial \theta }=-\frac{x}{{\left(\theta +x\right)}^{2}}$, we obtain the estimate of $\theta$ by substituting the $\left({x}_{i},{y}_{i}\right)$ data values into the single normal equation,

10 $$\frac{{x}_{1}}{{\left(\theta +{x}_{1}\right)}^{2}}\left({y}_{1}-\frac{{x}_{1}}{\theta +{x}_{1}}\right)+\dots +\frac{{x}_{4}}{{\left(\theta +{x}_{4}\right)}^{2}}\left({y}_{4}-\frac{{x}_{4}}{\theta +{x}_{4}}\right)=0$$

This expression is a nonlinear equation in the model function parameter, $\theta$. The “uniroot” function in R (see the Supplementary Information) is used to obtain the LSE which here is $\widehat{\theta }=5.8698$. ■

#### Example 2 (continued)

For the two-parameter vector $${\varvec{\theta}}=\left(\begin{array}{c}\alpha \\ \theta \end{array}\right)$$, the model function for this illustration is $\eta \left(x,{\varvec{\theta}}\right)=\alpha \left(1-\frac{x}{2\theta }\right)$ and so the sum of squares function is $S\left({\varvec{\theta}}\right)={\sum }_{i=1}^{6}{\left({y}_{i}-\alpha \left(1-\frac{{x}_{i}}{2\theta }\right)\right)}^{2}$. Differentiating with respect to the model parameters gives the expressions,

11 $$\frac{\partial S({\varvec{\theta}})}{\partial \alpha }=-2{\sum }_{i=1}^{6}\left(1-\frac{{x}_{i}}{2\theta }\right)\left({y}_{i}-\alpha \left(1-\frac{{x}_{i}}{2\theta }\right)\right)$$

and

12 $$\frac{\partial S({\varvec{\theta}})}{\partial \theta }=-2{\sum }_{i=1}^{6}\frac{\alpha {x}_{i}}{2{\theta }^{2}}\left({y}_{i}-\alpha \left(1-\frac{{x}_{i}}{2\theta }\right)\right)$$

When these two equations are set equal to zero, we obtain the two nonlinear normal equation system in two unknowns ($\widehat{\alpha }$ and $\widehat{\theta }$), i.e., the normal equations. For the data used in this example, the numerical algorithm yields the LSEs $\widehat{\alpha }=32.639$ and $\widehat{\theta }=22.327$ as reported previously. ■

The manner of finding standard errors for model function parameters is similar to that used for linear models but for nonlinear models it is based on the following approximation. The first order (and also asymptotic) variance–covariance matrix associated with the LSE parameter vector estimate $\widehat{{\varvec{\theta}}}$ is ${s}^{2}{({{\varvec{X}}}^{T}{\varvec{X}})}^{-1}$ where the mean-square error (MSE) is ${s}^{2}=\frac{S\left(\widehat{{\varvec{\theta}}}\right)}{n-p}$ and the Jacobian matrix ${\varvec{X}}$ is given in Eq. (7). The diagonal elements of ${s}^{2}{({{\varvec{X}}}^{T}{\varvec{X}})}^{-1}$ are the squares of the standard errors (SEs) of the parameter estimates $\widehat{{\varvec{\theta}}}$. Note that for linear models, these standard errors are exact but for nonlinear models, they are based on a first-order (or large-sample) approximation.

To illustrate these results for Example 3, since here ${s}^{2}=\frac{S\left(\widehat{{\varvec{\theta}}}\right)}{n-p}=\frac{0.02217}{3}=0.00739$ and ${{\varvec{X}}}^{T}{\varvec{X}}$ is the scalar ${\sum }_{i=1}^{4}\frac{{x}_{i}^{2}}{{\left(\widehat{\theta }+{x}_{i}\right)}^{4}}=0.004542$, the standard error associated with $\widehat{\theta }=5.8698$ is $SE=\sqrt{\frac{0.00739}{0.004542}}=\sqrt{1.6269}=1.2755$. Analogous results can be obtained for Examples 1 and 2 but in these cases, since ${{\varvec{X}}}^{T}{\varvec{X}}$ is of dimension $2\times 2$, matrix inversion is used to find ${\left({{\varvec{X}}}^{T}{\varvec{X}}\right)}^{-1}$ and thereby the corresponding standard errors associated with the LSE parameter estimates.

### Parameter Estimation: Interval Estimates

In statistical methodology, confidence interval strategies and methodologies can be obtained by “inverting” a test statistic. For example, in many single parameter situations (such as for a paired t-tests or regression through the origin), the null hypothesis ${H}_{0}:\theta ={\theta }_{0}$ can be tested using the test statistic,

13 $$t=\frac{\widehat{\theta }-{\theta }_{0}}{SE}$$

Here, $\widehat{\theta }$ and $SE$ are the corresponding parameter estimate and standard error. Under certain normal theory assumptions, this so-called Wald test statistic follows a t-distribution with $n-1$ degrees of freedom (Wald 1943). The test statistic is rearranged and solved for ${\theta }_{0}$ to produce the associated Wald $\left(1-\alpha \right)100\%$ confidence interval for $\theta$, viz, $\widehat{\theta }\pm {t}_{\left(\alpha /2\right),(n-1)}\times SE$. Here, ${t}_{\left(\alpha /2\right),(n-1)}$ is the t-distribution quantile with $n-1$ degrees of freedom which puts area $\alpha /2$ in both the lower and the upper tails. Similarly, for the p-dimensional parameter case where $p>1$, the corresponding Wald confidence interval (WCI) for parameter ${\theta }_{i}$, $i=\mathrm{1,2},\dots ,p$, is

14 $${\widehat{\theta }}_{i}\pm {t}_{\left(\alpha /2\right),\left(n-p\right)}\times {SE}_{i}$$

As noted in the previous section, ${SE}_{i}$ is the square root of the ${i}^{th}$ diagonal element of the variance–covariance matrix ${s}^{2}{\left({{\varvec{X}}}^{T}{\varvec{X}}\right)}^{-1}$. The degrees of freedom of the $t$ quantile here is $n-p$.

For this one-parameter hypothesis test ${H}_{0}:\theta ={\theta }_{0}$, a rival test statistic to the above Wald test statistic is the likelihood-based F-statistic,

15 $$F=\frac{S\left({\theta }_{0}\right)-S\left(\widehat{\theta }\right)}{{s}^{2}}$$

When ${H}_{0}$ is true, this test statistic has the F distribution with $1$ and $(n-1)$ degrees of freedom. Inverting this test statistic gives the likelihood-based confidence interval (LBCI) which, since ${s}^{2}=\frac{S\left(\widehat{{\varvec{\theta}}}\right)}{n-1}$ here, consists of the values of $\theta$ such that

16 $$S\left(\theta \right)=S\left(\widehat{\theta }\right)\left(1+\frac{{F}_{1,(n-1)}}{n-1}\right)=S\left(\widehat{\theta }\right)\left(1+\frac{{t}_{n-1}^{2}}{n-1}\right)$$

In this expression, $S\left(\theta \right)={\sum }_{i=1}^{n}{\left({y}_{i}-\eta \left({x}_{i},{\varvec{\theta}}\right)\right)}^{2}$ is the sum of squares function introduced and discussed previously. Note that Eq. (16) also makes use of the fact that the square of a t-quantile with $k$ degrees of freedom is equal to the corresponding F-quantile with $1$ and $k$ degrees of freedom. Once the data have been obtained and used to estimate the single model parameter $\theta$, and once the confidence level has been set, the right-hand side of Eq. (16) is a positive number. And, per this equation, finding the values of $\theta$ for which $S\left(\theta \right)$ is equal to that positive number is again a nonlinear root-finding undertaking which generally uses numerical methods to solve. The following example provides an illustration of these Wald and likelihood-based confidence interval methodologies.

#### Example 3 (continued).

As reported in Sect. 3.2, for the $n=4$ simulated data points and the one-parameter model function given in Eq. (9), the LSE parameter estimate and standard error are $\widehat{\theta }=5.8698$ and $SE=1.2755$ respectively. The 95% t-quantile, obtained using the R command, qt(0.975,3), is 3.1824, and so the 95% (Wald) WCI is $5.8698\pm 3.1824\times 1.2755$ or$(1.8106 , 9.9291)$. Finding the (likelihood-based) LBCI is a little more challenging since it is the interval of $\theta$ values between the interval end-point values for which$S\left(\theta \right)=S\left(\widehat{\theta }\right)\left(1+\frac{{t}_{n-1}^{2}}{n-1}\right)=0.09702$. Using the “uniroot” R function employed in the Supplementary Information, the LBCI here is the values of $\theta$ in the interval$(2.9960 , 12.7122)$. This simple illustration demonstrates a pronounced difference between these two types of 95% confidence intervals for these data. For example, the test that ${H}_{0}:\theta =11$ would be retained using the likelihood method but rejected using the Wald approach. The opposite conclusion would follow in testing${H}_{0}:\theta =2$. We emphasize that whereas these two types of intervals coincide exactly for linear models, this is clearly not the case for nonlinear models. ■

We next demonstrate how the Wald approach supplies an approximation to the likelihood approach. Applying the first-order Taylor series approximation of the one parameter model function about $\widehat{\theta }$ and substituting this approximation in the sum-of-squares function, we obtain

$$\eta \left(x,\theta \right)\approx \eta \left(x,\widehat{\theta }\right)+\frac{\partial \eta \left(x,\widehat{\theta }\right)}{\partial \theta }\left(\theta -\widehat{\theta }\right)$$

$${y}_{i}-\eta \left({x}_{i},\theta \right)\approx {y}_{i}-\eta \left({x}_{i},\widehat{\theta }\right)-\frac{\partial \eta \left({x}_{i},\widehat{\theta }\right)}{\partial \theta }\left(\theta -\widehat{\theta }\right)$$

So,

17 $${\sum }_{i=1}^{n}{\left({y}_{i}-\eta \left({x}_{i},\theta \right)\right)}^{2}\approx {\sum }_{i=1}^{n}{\left({y}_{i}-\eta \left({x}_{i},\widehat{\theta }\right)\right)}^{2}+\left\{{\sum }_{i=1}^{n}{\left[\frac{\partial \eta \left({x}_{i},\widehat{\theta }\right)}{\partial \theta }\right]}^{2}\right\}{\left(\theta -\widehat{\theta }\right)}^{2}$$

The last line in Eq. (17) follows from the penultimate line by squaring, summing over $i$ from $1$ to $n$, and noting that the cross-product term is zero by the normal equation. This last line of Eq. (17) shows that subject to the assumed first-order approximation, $S\left(\theta \right)$ is approximately equal to the constant $S\left(\widehat{\theta }\right)$ plus a quadratic expression in $\theta$. When combined with Eq. (16), this provides the Wald interval given in Eq. (14). This demonstrates that Wald intervals are quadratic approximations to the true sum of squares function and result from an initial first-order approximation. These results are illustrated as follows.

#### Example 3 (continued).

**Fig. 2:**
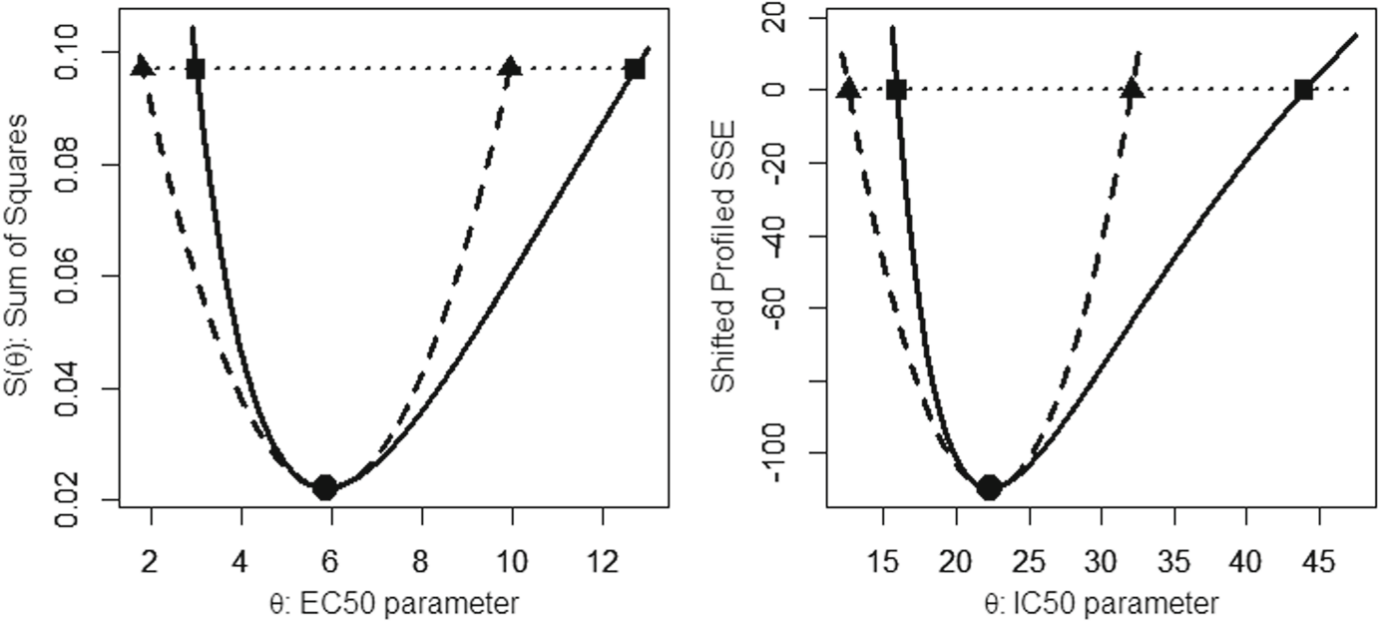
Sum of squares plots. Left panel: For Example 3 data, Michaelis–Menten-like sum of squares plot (solid curve) and Wald approximation (dashed parabola), least-squares parameter estimate (filled circle), LBCI endpoints (filled squares) and WCI endpoints (filled triangles). Right panel: For Example 2 data, laetisaric acid shifted profiled sum of squares plot (solid curve) and Wald approximation (dashed parabola), least-squares parameter estimate (filled circle), PLCI endpoints (filled squares) and WCI endpoints (filled triangles)

For the given data, the sum of squares function $S\left(\theta \right)$ is plotted in the left panel of Fig. 2 using the solid curve and where the filled circle is the point$(\widehat{\theta },S\left(\widehat{\theta }\right))=(5.8698, 0.02217)$. Also plotted is the horizontal cut line at $y=S\left(\widehat{\theta }\right)\left(1+\frac{{t}_{n-1}^{2}}{n-1}\right)=0.09702$ obtained from Eq. (16). The intersection of $S\left(\theta \right)$ and the cut line gives the endpoints of the LBCI,$(2.9960 , 12.7122)$, as indicated by the filled squares in the left panel. Also plotted is the (Wald) quadratic approximation as the dashed parabola; the intersection of this quadratic approximation and the cut line gives the endpoints of the WCI,$(1.8106 , 9.9291)$. Although the exact shape of the sum of squares function $S\left(\theta \right)$ is not relevant to the practitioner, what is important is that the Wald method is based on a linear approximation (which when squared gives the parabola) and that the two methods generally differ for nonlinear models.

Before leaving this example, two other comments are important to note. First, notice that the LSE point estimate is the same whether we use the true $S\left(\theta \right)$ function or the quadratic approximation. This is to be expected since the linear approximation takes place at $\theta =\widehat{\theta }$, where the two functions are equal. Second, using Eq. (16), the cut-line for the 90% confidence intervals is easily computed to be $y=0.06310$. At this lower height (and lower confidence level), note that the difference between the Wald and likelihood-based intervals is less pronounced. Thus in general, the higher the confidence level, the greater will generally be the divergence between the two intervals for nonlinear models. ■

As noted in Eq. (14), Wald confidence interval methods for the multi-parameter case of $p>1$ model parameters are straightforward. Methods for obtaining likelihood intervals involve the technique of parameter profiling which we now discuss. First, partition the p-dimensional parameter vector ${\varvec{\theta}}$ as $$\left(\begin{array}{c}{{\varvec{\theta}}}_{1}\\ {{\varvec{\theta}}}_{2}\end{array}\right)$$ where ${{\varvec{\theta}}}_{1}$ contains ${p}_{1}$ model parameters and ${{\varvec{\theta}}}_{2}$ contains ${p}_{2}$ model parameters so that ${p}_{1}+{p}_{2}=p$. To test the subset null hypothesis ${H}_{0}:{{\varvec{\theta}}}_{2}={{\varvec{\theta}}}_{20}$, the likelihood-based F test statistic (i.e., the counterpart of Eq. (15)) is

18 $$F=\frac{\left(S\left({\widetilde{{\varvec{\theta}}}}_{1},{{\varvec{\theta}}}_{20}\right)-S\left(\widehat{{\varvec{\theta}}}\right)\right)/{p}_{2}}{{s}^{2}}=\frac{\left(S\left({\widetilde{{\varvec{\theta}}}}_{1},{{\varvec{\theta}}}_{20}\right)-S\left(\widehat{{\varvec{\theta}}}\right)\right)/{p}_{2}}{S\left(\widehat{{\varvec{\theta}}}\right)/(n-p)}$$

Under the null hypothesis, this test statistic follows the ${F}_{{p}_{2},(n-p)}$ distribution, that is, the F distribution with ${p}_{2}$ and $(n-p)$ degrees of freedom. In Eq. (18), ${\widetilde{{\varvec{\theta}}}}_{1}$ minimizes $S({\varvec{\theta}})$ subject to the constraint ${H}_{0}:{{\varvec{\theta}}}_{2}={{\varvec{\theta}}}_{20}$. This technique of removing so-called nuisance parameters (i.e., ${{\varvec{\theta}}}_{1}$ here) by constrained optimization is the mentioned profiling technique. Note that the restricted (constrained) estimate ${\widetilde{{\varvec{\theta}}}}_{1}$ is in general not equal to the unrestricted (LSE) estimate ${\widehat{{\varvec{\theta}}}}_{1}$. Furthermore, since our interest is in obtaining a confidence interval instead of a region, let ${p}_{2}=1$ so that ${\theta }_{2}$ is the single parameter of interest. Then, the result in Eq. (18) is

19 $$\frac{S\left({\widetilde{{\varvec{\theta}}}}_{1},{\theta }_{20}\right)-S\left(\widehat{{\varvec{\theta}}}\right)}{S\left(\widehat{{\varvec{\theta}}}\right)\vphantom{^{T^{T}}}/(n-p)} \sim {F}_{1,(n-p)}$$

Inverting this expression gives the profile likelihood confidence interval (PLCI) as the set of ${\theta }_{2}$ values which solve the (typically nonlinear) equation

20 $$S({\widetilde{{\varvec{\theta}}}}_{1},{\theta }_{2})=S\left(\widehat{{\varvec{\theta}}}\right)\left(1+\frac{{F}_{\alpha ,1,(n-p)}}{n-p}\right)=S\left(\widehat{{\varvec{\theta}}}\right)\left(1+\frac{{t}_{\left(\alpha /2\right),(n-p)}^{2}}{n-p}\right)$$

We underscore that ${\widetilde{{\varvec{\theta}}}}_{1}={\widetilde{{\varvec{\theta}}}}_{1}({\theta }_{2})$ in Eq. (20) is the value of the remaining $(p-1)$-vector ${{\varvec{\theta}}}_{1}$ in $${\varvec{\theta}}=\left(\begin{array}{c}{{\varvec{\theta}}}_{1}\\ {\theta }_{2}\end{array}\right)$$ that minimizes $S({\varvec{\theta}})$ subject to the constraint that ${\theta }_{2}$ is the given fixed value. In certain instances, algebraic results can be derived to obtain the ${\widetilde{{\varvec{\theta}}}}_{1}$ vector in closed-form, but in the general situation, numerical methods are required.

Direct comparison of the LBCI expression in Eq. (16) with the PLCI expression in Eq. (20) highlights the fact that both approaches use root finding methods to find the corresponding confidence intervals. But the PLCI equation also involves constrained optimization to remove the remaining (so-called ‘nuisance’) parameters. The following example illustrates the PLCI method for a situation where an exact algebraic result is available.

#### Example 2 (continued).

As noted above, the key parameter in this example is the ${IC}_{50}$ parameter, $\theta$, so that the intercept parameter $\alpha$ is treated as a nuisance parameter. That is, $\alpha$ is a parameter which must be estimated but which is not the main focus of the study. The intercept $\alpha$ is profiled out by fixing $\theta$ and setting to zero only the partial derivative $\frac{\partial S({\varvec{\theta}})}{\partial \alpha }$ in Eq. (11). This gives the profiled (conditional) parameter estimate,

21 $$\widetilde{\alpha }=\widetilde{\alpha }\left(\theta \right)=\frac{{\sum }_{i=1}^{6}{y}_{i}\left(1-\frac{{x}_{i}}{2\theta }\right)}{{\sum }_{i=1}^{6}{\left(1-\frac{{x}_{i}}{2\theta }\right)}^{2}}$$

This profiled parameter estimate is then substituted into the sum of squares function to obtain the profiled sum of squares function,

22 $$S\left(\theta \right)=S\left(\widetilde{\alpha },\theta \right)={\sum }_{i=1}^{6}{\left({y}_{i}-\widetilde{\alpha }\left(1-\frac{{x}_{i}}{2\theta }\right)\right)}^{2}$$

For the given data, this profiled sum of squares function is plotted in the right panel of Fig. 2 using the solid curve and where the filled circle is the point$(\widehat{\theta },S\left(\widehat{{\varvec{\theta}}}\right))=(22.327, -109.5497)$. For ease in computations, this profiled sum of squares function has been shifted down here by the amount $S\left(\widehat{{\varvec{\theta}}}\right)\left(1+\frac{{t}_{n-2}^{2}}{n-2}\right)$ so the horizontal cut line of Eq. (20) is then $y=0$. The intersection of the shifted profiled $S\left(\theta \right)$ function and the horizontal cut line gives the endpoints of the PLCI,$(15.9176 , 43.9640)$, as indicated by the filled squares in the figure. Also plotted is the (Wald) quadratic approximation as the dashed parabola; the intersection of this quadratic approximation and the cut line gives the endpoints of the WCI,$(12.5786 , 32.0756)$. ■

The plots in the two panels of Fig. 2 look very similar but note that the plots in the left panel are for the sum of squares function, whereas in the right panel they correspond to the profiled sum of squares function. Regardless, in both cases, the Wald and likelihood curves and intervals are observed to differ appreciably.

#### Examples 1 and 2 (continued).

**Fig. 3:**
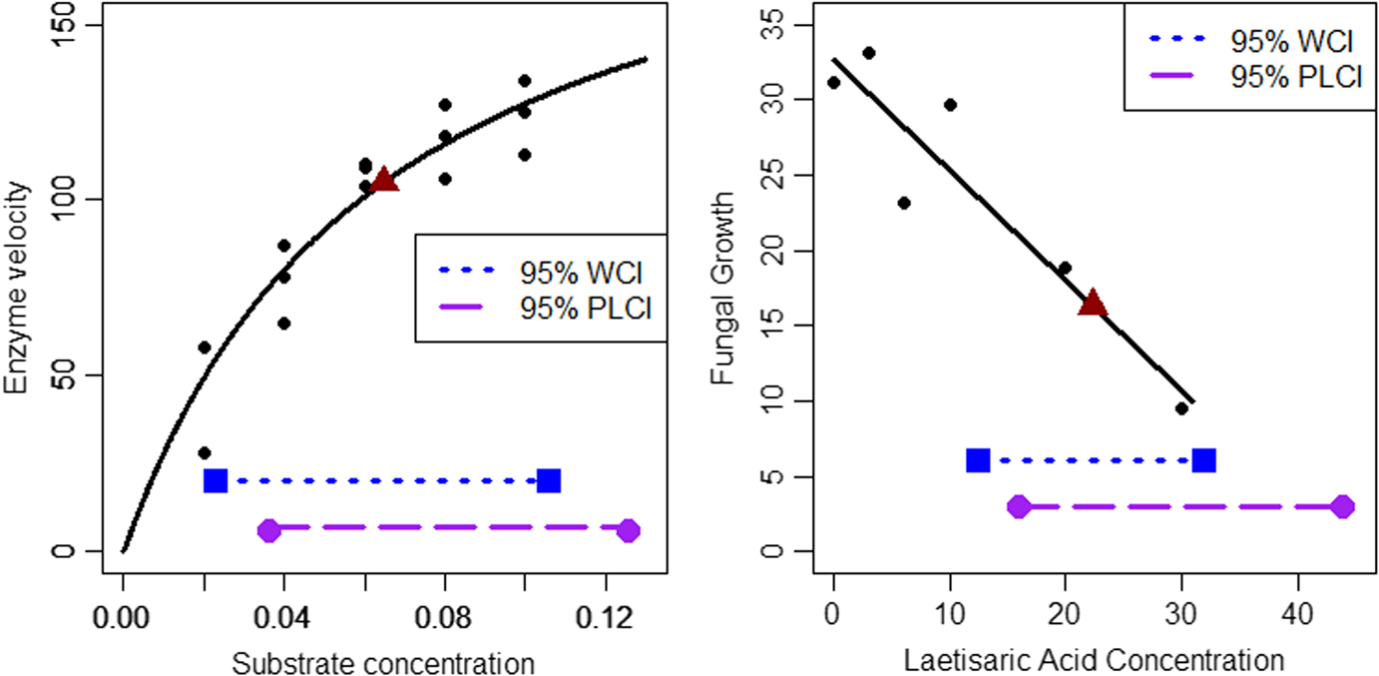
Two motivating example plots with confidence intervals. Left panel: Plot of simulated data (small, filled circles), fitted two-parameter Michaelis–Menten model function, estimated ${EC}_{50}$ point (large, filled triangle), 95% Wald confidence interval (WCI) (short-dashed line segment between large, filled squares) and 95% profile likelihood confidence interval (PLCI) (long-dashed line segment between large, filled circles). Right panel: Plot of fungal growth data (small, filled circles), fitted line, estimated ${IC}_{50}$ point (large, filled triangle), 95% Wald confidence interval (WCI) (short-dashed line segment between large, filled squares) and 95% profile likelihood confidence interval (PLCI) (long-dashed line segment between large, filled circles). The confidence intervals are spanned by the horizontal lines at the bottom of the panels

To summarize the previous findings for Sect. 2’s motivating examples and to introduce the topic of the next section, we briefly return to these two 2-parameter examples now displayed in the panels of Fig. 3.

In both of these examples, the Wald intervals are (by construction) symmetric about the respective LSEs whereas the profile likelihood intervals are shifted to the right. ■

We next turn to giving practical reasons for preferring one of these confidence interval methods over the other. Then, in Sect. 3.5, we discuss nonlinear model selection and computational algorithms.

### Deciding Which Confidence Interval Method is Preferred and Why

For nonlinear model function parameter estimation, F-statistic likelihood-based confidence intervals are generally preferred to Wald methods for several important reasons. These reasons, discussed further below, have been underscored by several notable works (Bates and Watts 2007; Clarke 1987; Cook and Witmer 1985; Donaldson and Schnabel 1987; Evans et al. 1996; Faraggi et al. 2003; Haines et al. 2004; Pawitan 2013; Peddada and Haseman 2005; Ratkowsky 1983; Seber and Wild 1989). As regards the notation used here, Wald confidence intervals (WCIs) are those given in Eq. (14) and likelihood confidence intervals are either likelihood-based confidence intervals (LBCIs) in the one-parameter setting such as in Eq. (16) or profile-likelihood confidence intervals (PLCIs) for the multiparameter setting such as for the two 2-parameter motivating examples displayed in Fig. 3 and as in Eq. (20).

There are several important reasons why likelihood confidence interval methods (LBCIs and PLCIs) are preferred over WCIs for nonlinear modelling. One reason is that likelihood intervals generally have much better agreement between nominal (i.e., assumed) confidence levels and actual confidence levels. Several works (Clarke 1987; Donaldson and Schnabel 1987; Evans et al. 1996; Faraggi et al. 2003; Ratkowsky 1983; Seber and Wild 1989) have used computer simulations to demonstrate that, provided the model/data’s intrinsic curvature (discussed in Appendix A.1) is reasonably low, likelihood intervals typically demonstrate good agreement between the chosen nominal (e.g., 95%) and the actual confidence level. Results for Wald intervals, however, can be quite disappointing. For example, simulation studies for some reported homoscedastic normal nonlinear models have found that the “observed coverage for a nominally 95% [Wald] confidence interval is as low as 75.0%, 44.0%, and 10.8%” (Donaldson and Schnabel 1987, p.76), depending on the data and chosen model function.

The superior coverage of likelihood methods is not surprising and is to be expected since for homoscedastic normal nonlinear models, the likelihood test statistics given in Eqs. (15), (18), and (19)—and the associated likelihood confidence intervals—are exact or very nearly so. Theoretical results show that the only difference between these F-statistic results and exact results (including p-values and coverage probabilities) depends upon the model’s intrinsic curvature (discussed in Appendix A.1). Further, intrinsic curvature is often negligible for nonlinear models in practice (Bates and Watts 2007; Clarke 1987; Ratkowsky 1983; Seber and Wild 1989). Wald confidence intervals, on the other hand, are also affected by so-called parameter-effects curvature (also discussed in Appendix A.1), which can be appreciable for many nonlinear model-dataset situations.

Another reason likelihood methods are preferred is that they more accurately reflect the information in the data. For example, for the two models and datasets of Fig. 3, the WCIs are symmetric whereas the PLCIs are shifted to the right. Since most of the datapoints (e.g., five of the six data points in the right panel) lie to the left of the estimated ${IC}_{50}$, the relative amount of ‘information’ in the data about the ${IC}_{50}$ parameters is higher on the left of the ${IC}_{50}$ estimate and lower on the right side. So the PLCIs are more reasonable for these datasets and models since less information is contained in the data on the right side of the estimated parameters and so the PLCIs extend further on the right-hand side. More generally, since WCIs for nonlinear model parameters are always symmetric and PLCIs can and often are asymmetric, PLCIs can more accurately reflect any information/precision imbalances in the data regarding specific parameter values.

In sum, for all practical purposes, the F-based likelihood methods used here are essentially exact (see Appendices A.1 and A.2). On the other hand, Wald methods for nonlinear model parameters are based on the asymptotic (valid for large-sample) normality of the model parameter estimate. Since this approximation breaks down for many nonlinear models with small-to-moderate datasets, these Wald methods should only be used with caution or avoided altogether—and this includes the commonly reported Wald *p*-values given by some popular statistical software packages.

### Nonlinear Model Function Selection and Computational Methods

As regards model function selection, our preference is to use mechanistic models instead of empirical models whenever possible. Mechanistic models are those chosen based on the subject-matter knowledge of the relevant system or phenomenon under study, whereas empirical models are those often chosen based on providing a good fit to the study data. In early-stage studies of two or more quantitative factors, empirical modelling sometimes includes response surface modelling such as quadratic (or higher-order) polynomial fitting. As expert knowledge of the system grows, focus often shifts to nonlinear modelling, such as using dose response or similar (nonlinear) model(s).

Mechanistic nonlinear model functions are sometimes based on a system of one or more differential equation(s). These are equations that model rates of change in the given system. These so-called compartmental models are popular in fields including chemical kinetics, ecology, pharmacology (including pharmacokinetics and pharmacodynamics) and toxicology (Bates and Watts 2007; Seber and Wild 1989). For example, the exponential decay model function for the population of an ecosystem at time $t$,

23 $$P\left(t\right)={P}_{0}{e}^{-rt},$$

is a solution of the differential equation (with given initial condition),

24 $$\frac{dP(t)}{dt}=-rP(t), P\left(0\right)={P}_{0}$$

This differential equation posits that the rate of decrease in the population at time $t$ is proportional to the size of the population at that time. For another example, if the rate of change of the size of a biological culture is assumed to grow rapidly at first up to a point and then decrease (e.g., with increased competition), a commonly-assumed differential equation with ‘half-life’ condition is,

25 $$\frac{dP(t)}{dt}=\frac{{\theta }_{3}}{{\theta }_{1}}P(t)\left({\theta }_{1}-P(t)\right), P\left({\theta }_{2}\right)=\frac{{\theta }_{1}}{2}$$

A solution of this differential equation is the three-parameter, normally distributed logistic growth model function,

26 $$P(t)=\frac{{\theta }_{1}}{1+{e}^{-{\theta }_{3}\left(t-{\theta }_{2}\right)}}$$

Other models, such as the intermediate-product model in pharmacokinetics (Bates and Watts 2007), involve systems of two or more differential equations.

Parameter estimation for nonlinear models is generally achieved using iterative methods such the Newton–Raphson method (Ratkowsky 1983) or some variant thereof. This method involves successively substituting linear approximations such as those used in Eq. (17) into the sum of squares function and/or normal equations. This process is repeated “until convergence,” meaning until the changes in the objective function between iterations are below some chosen threshold. These computational algorithms have been implemented into the NLIN, NLP and NLMIXED procedures in SAS, the “nls,” “nlmer” and “gnm” functions in R, and other software packages such as GAUSS, Minitab, PRISM, STATA, etc. Paramount to this process is the necessity of well-chosen starting points, which is best achieved by first understanding the roles of the individual model function parameters and plotting the given data. Further details of computation aspects for nonlinear modelling can be found in nonlinear regression texts (Bates and Watts 2007; Ratkowsky 1983; Seber and Wild 1989).

## Additional Nonlinear Illustrations

The following examples further serve to illustrate the wide-ranging applications of nonlinear modelling and are included for readers wishing for additional examples.

### Example 4

**Fig. 4:**
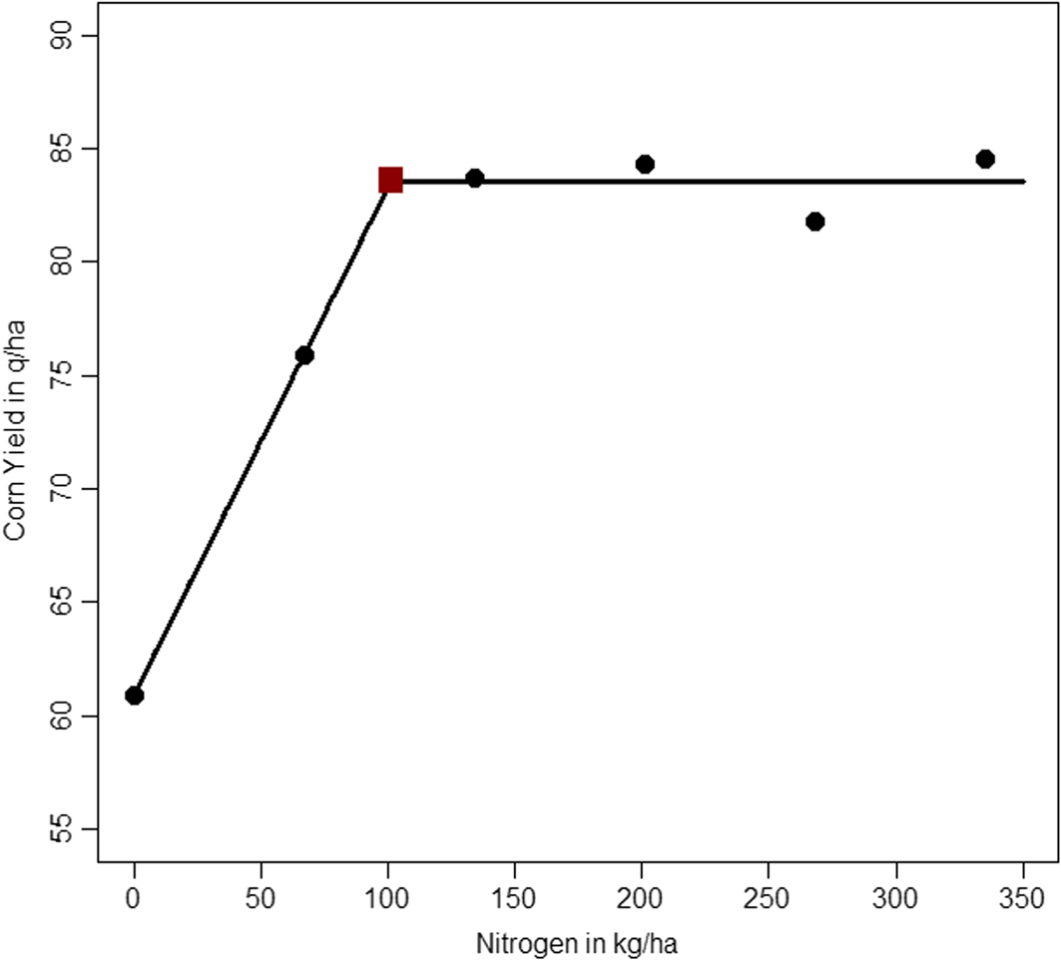
Simple Spline Fit. Corn yield versus nitrogen fertilizer data (six filled circles), fitted linear-plateau segmented curve, and estimated knot or join-point (filled square)

The nonlinear model discussed here is a segmented regression function model (also called a broken-stick, piecewise, or change-point model). This model function is used in data science and application fields such as agronomy, economics, engineering, environmental studies, and medicine (Seber and Wild 1989). The data examined here (Anderson and Nelson 1975) and graphed in Fig. 4 relate average corn yields (the outcome variable) to the amount of nitrogen fertilizer applied (the input variable). Following the authors, the linear-plateau segmented model is fitted here, and the corresponding fitted linear-plateau curve is also superimposed in the figure.

The linear-plateau model used here has parameter vector ${{\varvec{\theta}}}^{T}=\left(\alpha ,\beta ,\kappa \right)$ and is written

27 $$\eta \left(x,{\varvec{\theta}}\right)=\left\{\begin{array}{c}\alpha +\beta x,\mathrm{ for } \ x\le \kappa \\ {y}_{MAX}=\alpha +\beta \kappa ,\mathrm{ for } \ x>\kappa \end{array}\right.$$

This model function can also be written

28 $$\eta \left(x,{\varvec{\theta}}\right)=\left(\alpha +\beta x\right){ I}_{x\le \kappa }+\left(\alpha +\beta \kappa \right){ I}_{x>\kappa }$$

Here ${I}_{C}$ is an indicator function equal to one when condition $C$ in the subscript is true and equal to zero otherwise. In accordance with the underlying (agricultural) subject-matter reasoning used by the authors and as observing in the data plotted in Fig. 4, this chosen model is continuous at the unknown join or transition point $x=\kappa$. This is a nonlinear model since the transition point (also called a knot), $\kappa$, is a model parameter to be estimated, and for $x>\kappa$, the derivative $\frac{\partial \eta }{\partial \beta }=\kappa$ contains a model parameter.

Using the given R code (see the Supplementary Information), for these data the parameter estimates are $\widehat{\alpha }=60.90,\widehat{\beta }=0.22$ and $\widehat{\kappa }=101.28$, so the maximum corn yield is estimated to be ${\widehat{y}}_{MAX}=60.90+0.22\times 101.28=83.58$. The point $(\widehat{\kappa },{\widehat{y}}_{MAX})$ is the solid square plotted in Fig. 4. The 95% profile likelihood confidence interval for the transition point $\kappa$ is $(78.72, 143.48)$.

Although sound reasons were already given in Sect. 3.4 for avoiding the use of Wald methods, we re-emphasize that caution is given (Hinkley 1969; Seber and Wild 1989) to avoid using Wald-based methods for such segmented models since the required asymptotical normality approximation for $\widehat{\kappa }$ can often be quite poor; with such the small sample size of $n=6$, likelihood methods are instead recommended. This caution regarding WCIs should especially be borne in mind when using spline models with unknown knots such as in fitting smoothing splines and generalized additive models popular in the domains of predictive modelling and machine learning (James et al. 2021). ■

### Example 5.

Estimation of the ratio of two homoscedastic independent-sample normal means, referred to as the Fieller-Creasy problem (Cook and Witmer 1985; Creasy 1954; Fieller 1954), is the focus of this next illustration. For ${n}_{1}+{n}_{2}=n$, let ${y}_{11},{y}_{12},\dots ,{y}_{1{n}_{1}}$ denote the ${n}_{1}$ group 1 independent measurements and ${y}_{21},{y}_{22},\dots ,{y}_{2{n}_{2}}$ denote the ${n}_{2}$ group 2 independent measurements. The nonlinear Fieller-Creasy model function is written.

29 $$\eta \left(x,{\varvec{\theta}}\right)={\theta }_{1}x+{\theta }_{1}{\theta }_{2}\left(1-x\right)$$

In Eq. (29), $x=1$ for group 1 observations and $x=0$ for group 2 observations. Thus, ${{\varvec{\theta}}}^{T}=\left({\theta }_{1},{\theta }_{2}\right)$, ${\theta }_{1}={\mu }_{1}$ and ${\theta }_{2}={\mu }_{2}/{\mu }_{1}$. It follows that ${\theta }_{2}$ is the parameter of interest since it is the ratio of the two means and ${\theta }_{1}$ is the nuisance parameter; following Sect. 3.3, ${\theta }_{1}$ is removed by parameter profiling so as to find the PLCI for the ratio parameter, ${\theta }_{2}$.

To illuminate use of these methods here, we use the simulated dataset wherein the ${n}_{1}=3$ group 1 response values are ${y}_{1j}=3, 4, 5$ and the ${n}_{2}=8$ group 2 response values are ${y}_{2j}=6, 6, 7, 8, 8, 9, 10, 10$. Clearly, ${\widehat{\theta }}_{1}={\overline{y} }_{1}=4$ and, since ${\overline{y} }_{2}=8$, ${\widehat{\theta }}_{2}=8/4=2$. With $S(\widehat{{\varvec{\theta}}})=20$, the unbiased estimator of ${\sigma }^{2}$ is the mean-square error (MSE), ${s}^{2}=\frac{20}{11-2}=2.22$.

Using the results given in Appendix A.3, the $(1-\alpha )100\%$ Wald confidence interval (WCI) for ${\theta }_{2}$ is

30 $${\widehat{\theta }}_{2}\pm \frac{s{t}_{\left(\alpha /2\right),(n-2)}}{{\widehat{\theta }}_{1}}\sqrt{\frac{{n}_{1}+{n}_{2}{\widehat{\theta }}_{2}^{2}}{{n}_{1}{n}_{2}}}$$

Likewise, the Appendix A.3 results are used to show that the profile likelihood confidence interval (PLCI) for ${\theta }_{2}$ is

31 $$\frac{{\widehat{\theta }}_{2}}{\left(1-c\right)}\pm \frac{s{t}_{\left(\alpha /2\right),(n-2)}}{\left(1-c\right) {\widehat{\theta }}_{1}}\sqrt{\frac{{n}_{1}\left(1-c\right)+{n}_{2}{\widehat{\theta }}_{2}^{2}}{{n}_{1}{n}_{2}}}$$

In this expression, $c=\frac{{s}^{2}{t}_{\left(\alpha /2\right),(n-2)}^{2}}{{n}_{1}{\widehat{\theta }}_{1}^{2}}$, and $c$ is in the interval, $0<c<1$. Thus, the center of the PLCI, $\frac{{\widehat{\theta }}_{2}}{\left(1-c\right)}$, is shifted to the right of the center of the WCI, ${\widehat{\theta }}_{2}$.

For the given data, the 95% WCI is $(0.98, 3.02)$ and the 95% PLCI is $(1.30, 3.94)$. The rightward shift of the PLCI vis-à-vis the WCI is notable here. Also, whereas the Wald approach would retain equal means (i.e., value of one is retained for the ratio parameter, ${\theta }_{2}$), the likelihood approach clearly rejects this claim. ■

In the following continuation of Example 1, we extend the original illustration to comparing two curves and calculate a relative potency parameter based on the ratio methodology of the previous illustration.

### Example 1 continued.

**Fig. 5:**
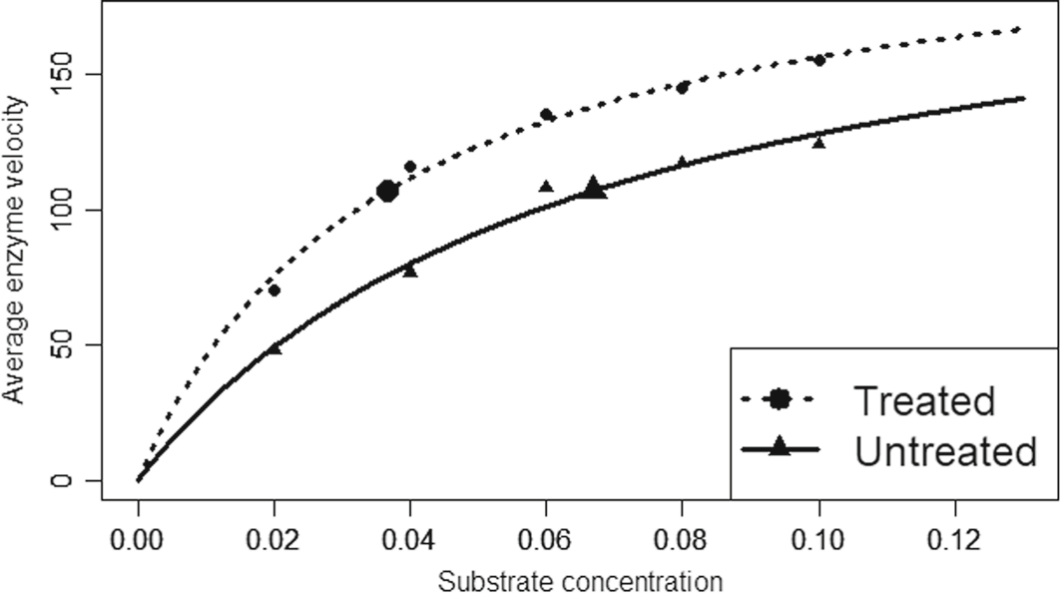
Treated and Untreated Enzyme Kinetic Model Fits. Average enzyme velocity versus concentration for antibiotic treated data (small, filled circles) with fitted common-upper-asymptote Michaelis–Menten curve (dashed curve) and untreated data (small, filled triangles) with fitted common-upper-asymptote Michaelis–Menten curve (solid curve). Also shown are estimated ${EC}_{50}$ points: treated (larger, filled circle) and untreated (larger, filled triangle)

The original Example 1 enzyme kinetic data analyzed previously and displayed in the left panel of Fig. 1 are for samples untreated with an antibiotic; the averages of the three (same concentration) replicates of these data are plotted in Fig. 5 using the small, filled triangles. In a spirit similar to other works (Bates and Watts 2007, p. 269), additional enzyme velocity measurements were made (also in triplicate) using the same substrate concentrations but for samples treated with the antibiotic. Averages of these replicates are also shown in Fig. 5 using the small, filled circles. (The fitted curves in the figure will be discussed below.) Using the relevant kinetics nonlinear modelling, researchers are interested in determining and quantifying the effect of the antibiotic on enzymatic activity.

To enable testing between the treated and untreated groups, the Michaelis–Menten model function of Eq. (1) is modified to fit both groups simultaneously using the model function,

32 $$\eta \left(x,{\varvec{\theta}}\right)=\frac{\left({\theta }_{1T}{D}_{T}+{\theta }_{1U}{D}_{U}\right)x}{\left({\theta }_{2T}{D}_{T}+{\theta }_{2U}{D}_{U}\right)+x}$$

In this expression, ${D}_{T}=1$ for samples in the treated group and ${D}_{T}=0$ for samples in the untreated group. Analogously, since ${D}_{U}=1-{D}_{T}$, one obtains ${D}_{U}=1$ for samples in the untreated group and ${D}_{U}=0$ for samples in the treated group. With ${{\varvec{\theta}}}^{T}=({\theta }_{1T},{\theta }_{1U},{\theta }_{2T},{\theta }_{2U})$, this model function expression is equal to $\eta \left(x,{\varvec{\theta}}\right)=\frac{{\theta }_{1T}x}{{\theta }_{2T}+x}$ for samples in the treated group and $\eta \left(x,{\varvec{\theta}}\right)=\frac{{\theta }_{1U}x}{{\theta }_{2U}+x}$ for samples in the untreated group, and so, as before, the ${\theta }_{1}$ and ${\theta }_{2}$ parameters are the respective upper asymptote and ${EC}_{50}$ parameters.

For the given data, the LSE parameter estimates are ${\widehat{\theta }}_{1T}=214.6, {\widehat{\theta }}_{1U}=209.9, {\widehat{\theta }}_{2T}=0.03712$ and ${\widehat{\theta }}_{2U}=0.06472$. The global test of one curve for both treatment groups, ${H}_{0}:{\theta }_{1T}={\theta }_{1U}, {\theta }_{2T}={\theta }_{2U}$, is soundly rejected with $F=25.76, p<0.0001$, but the claim of equal upper asymptotes, ${H}_{0}:{\theta }_{1T}={\theta }_{1U}, p=0.89$, is retained. (These results can be verified by running the R code in the Supplementary Information.)

The reduced two-group Michaelis–Menten model function with common upper asymptote is given by the expression,

33 $$\eta \left(x,{\varvec{\theta}}\right)=\frac{{\theta }_{1}x}{\left({\theta }_{2T}{D}_{T}+{\theta }_{2U}{D}_{U}\right)+x}=\frac{{\theta }_{1}x}{\left({\theta }_{2T}{D}_{T}+{\rho \theta }_{2T}{D}_{U}\right)+x}$$

Note that this expression is equal to $\eta \left(x,{\varvec{\theta}}\right)=\frac{{\theta }_{1}x}{{\theta }_{2T}+x}$ for the treated group and $\eta \left(x,{\varvec{\theta}}\right)=\frac{{\theta }_{1}x}{{\theta }_{2U}+x}$ for the untreated group, and the commonality of the upper asymptotes is noted. The connection between the right-hand expression of Eq. (33) is given by the relation,

34 $$\rho =\frac{{\theta }_{2U}}{{\theta }_{2T}}$$

The so-called relative potency parameter $\rho$ in Eq. (34) is the ratio of the respective ${EC}_{50}$ parameters, and it is in this context that this illustration mirrors Example 5; note too that by making it an explicit model function parameter, we can readily obtain an accurate (likelihood-based) confidence interval.

When the model function in Eq. (33) is fit to these data, the fitted curves are shown in Fig. 5 for the treated (top) and untreated (bottom) groups. For these data, the LSE estimate of $\rho$ is$\widehat{\rho }=1.8275$, so the substrate is approximately 1.8 times more potent for the treated group than for the untreated group. Further, the 95% PLCI for$\rho$,$(1.5274 , 2.2366)$, lies entirely above one, thereby establishing that the substrate is significantly more potent for the treated group than for the untreated group. ■

### Example 6.

**Fig. 6:**
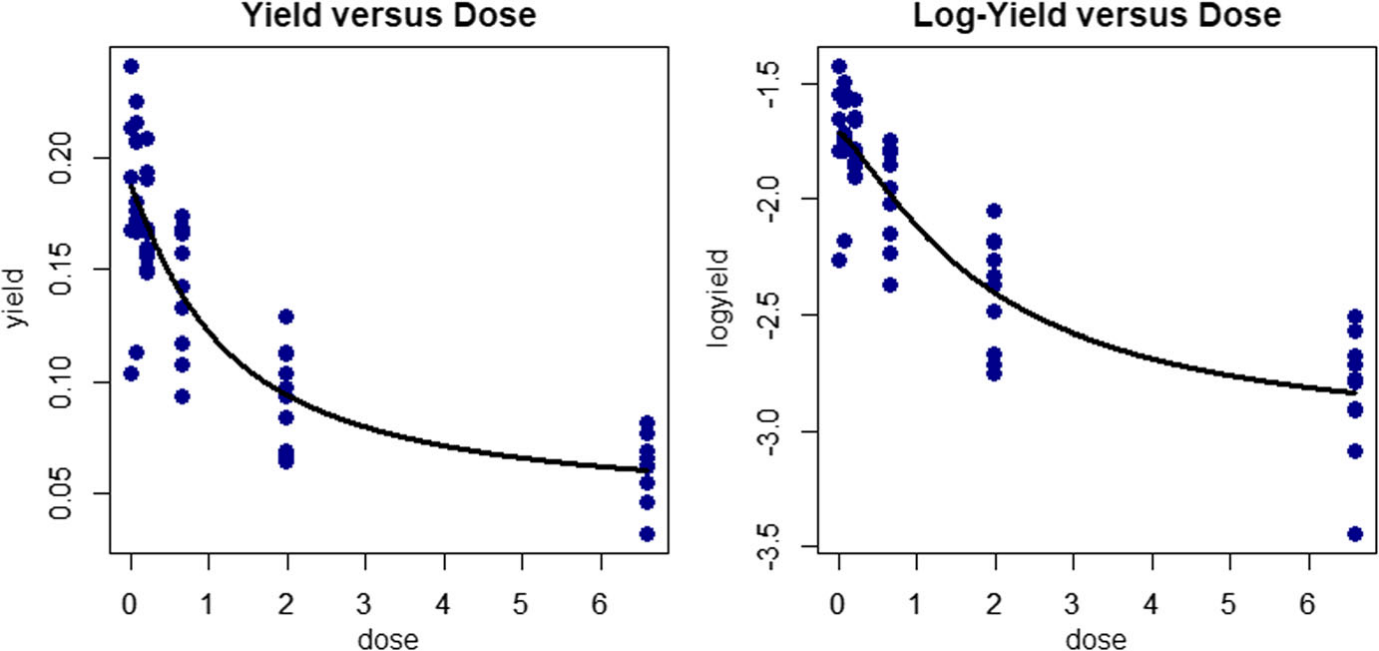
Wild Oat Dry Weight Dose Response Fits. Left panel: Original dry weight yield data plotted versus herbicide dose with heteroskedastic (variance function modelled) LL4 model fit (solid curve). Right panel: (Natural) Log-transformed dry weight yield data plotted versus herbicide dose with homoskedastic LL4 model fit (solid curve)

Examined here are dose–response data (Seefeldt et al. 1995) relating yield dry weight of biotype C wild oat *Avena fatua* (the response variable in g) to herbicide dose (the explanatory variable in kg ai/ha). These data are plotted in Fig. 6 with the raw data shown in the left panel and with the log-yield data plotted in the right panel. We use here the four-parameter log-logistic (LL4) model function (Seefeldt et al. 1995),

35 $$\eta \left(x,{\varvec{\theta}}\right)={\theta }_{2}+\frac{{\theta }_{1}-{\theta }_{2}}{1+{\left(\frac{x}{{\theta }_{3}}\right)}^{{\theta }_{4}}}, {{\varvec{\theta}}}^{T}=\left({\theta }_{1},{\theta }_{2},{\theta }_{3},{\theta }_{4}\right)$$

In this model function, which is also called the Hill equation or the Morgan-Mercer-Flodin family (Seber and Wild 1989), ${\theta }_{3}$ is the ${ED}_{50}$ (50% effective dose) parameter and ${\theta }_{4}$ is the slope parameter. For ${\theta }_{4}>0$, ${\theta }_{1}$ is the ‘upper asymptote,’ or the expected response when $x=0$ and ${\theta }_{2}$ is the lower asymptote or the expected response for very large dose (i.e., for $x\to \infty$). To establish that ${\theta }_{3}$ is the ${ED}_{50}$ parameter, note that when $x={\theta }_{3}$, the expected response is indeed $\frac{{\theta }_{1}+{\theta }_{2}}{2}$, the average of the two asymptotes.

In viewing the non-constant variance of the original data in the left panel of Fig. 5, we can fit the LL4 model function using log-yield as the response variable, and this fitted model function is superimposed as the solid curve in the right panel plot. Alternatively, after applying the log-transformation to both left and right sides of the equation, the log-yields could be fit using the logarithm of the LL4 model function. In this instance, the results in both cases are very similar. This practice of transforming the response variable (e.g., log-transformation here) with or without transformation of the model function, and then fitting the additive homoskedastic normal nonlinear model of Eq. (3), is quite commonly-used in practice. But, whether this is a sound practice depends on whether selected variance-stabilizing transformation (such as logarithm, square-root, etc.) is a good choice for the given dataset and model function. As such, we next consider an alternative strategy.

Although it falls outside of the constant-variance normal additive paradigm of Eq. (3), another option is to fit the additive LL4 model function to model the un-transformed responses and to also model the variances using a variance function such as

36 $$var\left({y}_{ij}\right)={\sigma }^{2}{\eta }^{\rho }\left({x}_{i},{\varvec{\theta}}\right)$$

In Eq. (36), in addition to the variance parameter, ${\sigma }^{2}$, an additional parameter, $\rho$, has been included as the power of the mean model function, $\eta \left(x,{\varvec{\theta}}\right)$. If $\rho =0$, then Eq. (36) reduces to the usual homoskedastic case where $var\left({y}_{ij}\right)={\sigma }^{2}$ of Eq. (3). Whenever $\rho >0$, this variance function holds that the variance (i.e., the spread of the data response values) decreases with the mean, and this behavior is indeed observed in the left panel of Fig. 5 since the variance of the responses is higher when the average yield is higher and lower when the average yield is lower. For the data plotted in the left panel of Fig. 6, the maximum-likelihood estimate of $\rho$ is $\widehat{\rho }=1.4707$, and the test of ${H}_{0}:\rho =0$ is rejected ($p<0.0001$). Using results in (Seber and Wild 1989), the estimate of $\widehat{\rho }\approx 1.5$ suggests that the fourth-root transformation (${y}^{1/4}$) may have been a better choice for these data than the log-transformation used above. For these data, however, since the results are very similar, the homoskedastic normal nonlinear fit shown in the right panel of Fig. 6 (for the log-transformed data) is deemed to be sufficient. ■

## Discussion and Final Thoughts

Before the advent of sufficient computing power and model-fitting methods, nonlinear models—often derived and based on sound expert-knowledge and theory—were historically fit by using linearization methods. This technique ignores the overall additive model structure given in Eq. (3) and the underlying model assumptions. For example, for the Michaelis–Menten model and function, $y=\frac{{\theta }_{1}x}{{\theta }_{2}+x}+\varepsilon$, if this expression is replaced with the approximation $y\approx \frac{{\theta }_{1}x}{{\theta }_{2}+x}$, algebraic manipulation leads to the expression $x/y\approx \left({\theta }_{2}/{\theta }_{1}\right)+\left(1/{\theta }_{1}\right)x$. With some further substitutions, the right-hand side of this expression is of the form $\alpha +\beta x$ and so linear models were then fit. Often, the resulting transformation introduced additional problems such as non-constant variance, lack-of-fit, and challenges in obtaining confidence intervals for the original model parameters. Although several authors (Currie 1982; Seber and Wild 1989) clearly warn against using such linearization methods, without introductory guides such as the current work, practitioners may not yet be aware of these problems.

In addition to the nonlinear regression methods and examples provided here, interested readers may wish to more fully explore topics such as further heteroskedastic (variance function) modelling, bioassay and synergy modelling (Lee et al. 2007; Lynch et al. 2016; Sims and O’Brien 2011; Straetemans et al. 2005; Tallarida 2000; Wheeler et al. 2006; White et al. 2019), multivariate, compartmental, and generalized nonlinear models, related experimental design considerations (Kim et al. 2021; O’Brien et al. 2010, O’Brien and Silcox 2021), and additional curvature examples (Seber and Wild 1989). Other notable recent application fields include the use of high-throughput dose response methods to evaluate compounds as potential antiviral drugs to treat COVID-19 patients (Chen et al. 2022) and modelling to assess enzymatic activity in viral proteins comparing SARS-CoV with SARS-CoV-2 (O’Brien et al. 2021).

### Electronic supplementary material

Below is the link to the electronic supplementary material.

Supplementary file1 (PDF 206 KB)

## Figures and Tables

**Fig. 7:**
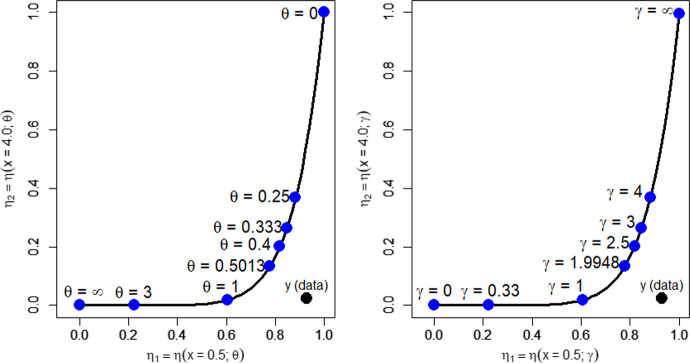
Illustration of Intrinsic ($IN$) and Parameter Effects ($PE$) Curvatures for Simple Exponential Example. Plots of the expectation surfaces (**E**) for one-parameter simple exponential model function/design for original ($\theta$) parameterization (left panel) and reciprocal ($\gamma =1/\theta$) parameterization (right panel). The data point is labelled “y (data)”. The selected points on each expectation surface are indexed by the indicated parameter values

**Fig. 8:**
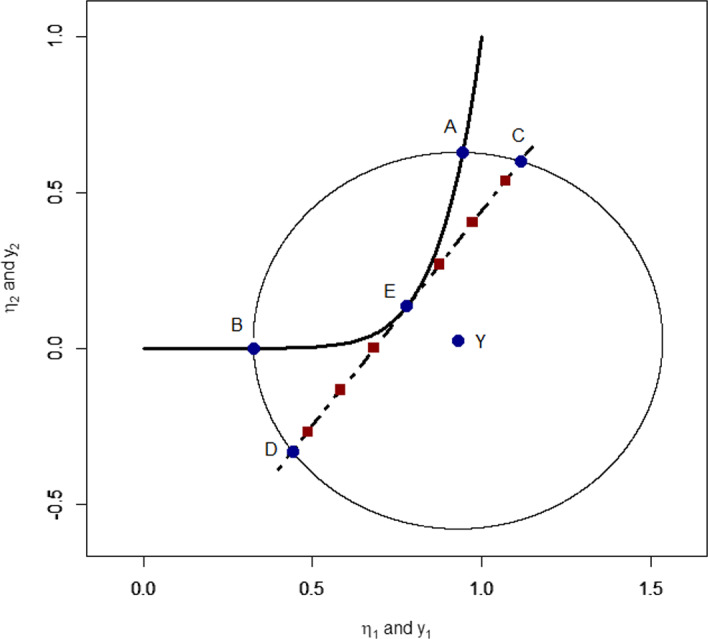
Distinguishing Wald and Likelihood Confidence Intervals. Plots of the expectation surface for the (original parameterization) simple exponential model and chosen design (curved surface **E**, the same as given in left panel of Fig. 7), data point (labelled Y), and point on **E** corresponding to the least-squares estimate $\widehat{\theta }=0.5013$ (labelled E). Also plotted are the tangent line approximation to **E** at E (the dot-dashed line segment) connecting points C and D, and the 80% confidence circle centered at Y. The points on **E** labelled A and B correspond to the likelihood confidence interval endpoints. The points on tangent line approximation corresponding to Wald confidence interval endpoints are labelled C and D. A regular-grid approximation on tangent line approximation is indicated using filled squares

**Fig. 9:**
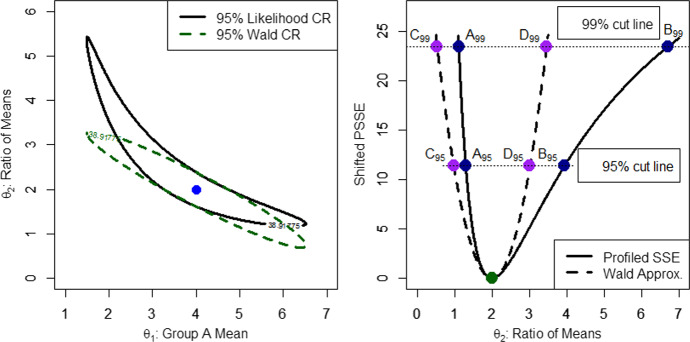
Confidence Regions and Intervals for the Fieller-Creasy Ratio of Means. Left panel: 95% joint confidence regions for Fieller-Creasy data: likelihood based region (solid region) and Wald approximate region (dashed ellipse). Right panel: profile likelihood sum-of-squares (SSE) curve for ratio-of-means parameter (solid curve), Wald approximation SSE curve (dashed parabola), 95% and 99% horizontal labelled cut-lines, and confidence intervals: 95 likelihood interval is $({A}_{95},{B}_{95})$, 95% Wald interval is $({C}_{95},{D}_{95})$ corresponding to lower circles, 99% likelihood interval is $({A}_{99},{B}_{99})$, and 99% Wald interval is $({C}_{99},{D}_{99})$ corresponding to upper circles

## Data Availability

All data used in this article is provided in the R code given in the Supplementary Information, and all needed permissions have been secured and granted.

## Appendix A.1. Why Wald and Likelihood Confidence Interval Methods Differ: Statistical Curvature and Model Function Reparameterizations

Although the question of why Wald and likelihood intervals differ is easy to pose, it is somewhat mathematically challenging to answer. As such, the discussion here is kept at the conceptual level. Further details are given in the references and readers wishing to understand these details (which are based on differential geometry and nonlinear statistical theory) are suggested to consult these outside sources.

Agreement or disagreement of Wald and likelihood-based confidence intervals can be assessed using so-called ‘nonlinearity’ or ‘curvature’ measures introduced in several works (Bates and Watts 2007; Clarke 1987; Haines et al. 2004; Ratkowsky 1983; Seber and Wild 1989). There are two main types of curvature measures: intrinsic (denoted $IN$) and parameter-effects (denoted $PE$) curvatures. Both $IN$ and $PE$ curvatures characterize nonlinear attributes of a model/dataset’s expectation surface. Although no hard-and-fast rules exist regarding these $IN$ and $PE$ measures, general rule-of-thumb guidelines have been given to decide when nonlinearity is problematic (Bates and Watts 2007). Furthermore, reparameterizing a model function can reduce the disagreement between likelihood and Wald intervals. This means that $PE$ curvature, which captures how parameters enter the model function, can be reduced; what’s left after all such parameter-effects curvature is removed is the intrinsic curvature ($IN$), which cannot be reduced and is inherent to the model function and data. Further, as pointed out in (Seber and Wild 1989, p.195), since likelihood methods are “invariant to reparameterizations, we can assume that a transformation has been found to remove the parameter-effects curvature,” and thus they are “only affected by intrinsic curvature, which is often negligible.” So, in practice, the main reason likelihood and Wald intervals differ is due to $PE$ curvature, and this in turn is related to the manner in which distances are calculated when finding confidence intervals. This distance metric is straightforward to assess for linear models where both $IN$ and $PE$ measures are zero, but is more challenging for nonlinear models. In light of the linear approximation used in Wald methods, the metric used for Wald distance and interval calculations is often inaccurate.

The following one-parameter illustration provides the development and illustration of the intrinsic ($IN$) and parameter-effects ($PE$) curvature measures. It also underscores the manner in which Wald confidence intervals approximate likelihood intervals and highlights how and when these intervals can diverge.

### Example 7.

The homoscedastic normal one-parameter simple exponential model function, used here and in drug studies involving pharmacokinetic one-compartment modelling (Bailer and Portier 1990), is given by the expression,

37 $$\eta \left(x, \theta \right)={e}^{-\theta x}, \theta >0$$

This one-parameter model function is fitted here to the simulated $n=2$-point data, $({x}_{1},{y}_{1})=(0.50, 0.93)$ and $({x}_{2},{y}_{2})=(4.0, 0.025)$. (Although it would be ill-advised to use such a small sample size in practice, choosing only two data points facilitates viewing the so-called one-dimensional expectation surface in this two-dimensional space.) These data and model yield the LSE/MLE $\widehat{\theta }=0.50$, the 80% likelihood-based confidence interval (LBCI), $(0.12, 2.25)$ and the 80% Wald confidence interval (WCI), $(-0.37, 1.37)$.

To appreciate how and why likelihood and Wald intervals differ, in the left panel of Fig. 7 is plotted the one-dimensional expectation surface (**E**) in the two-dimensional sample space for this model function and the given data (see also Bates and Watts 2007). Since here ${\eta }_{1}={e}^{-0.5\theta }$ and${\eta }_{2}={e}^{-4\theta }$, the expectation surface is given by the equation${\eta }_{2}={\eta }_{1}^{8}$. The expectation surface**E** is mapped out as $\theta$ ranges from $0$ to $\infty$ as indicated by the plotted $\theta$ values. Intrinsic curvature ($IN$) assesses the degree to which **E** deviates from a straight line here and from a plane or hyper-plane in the general $p>1$ case. Given the pronounced bending in **E** in Fig. 7, it is not surprising that the realized value of$IN$, calculated here to be$IN=1.44$, exceeds the suggested $0.30$ threshold value (Bates and Watts 2007). (Details of these calculations are omitted here and can be found in the references.) Thus, $IN$ is deemed to be “significant” in this instance. We again underscore that for linear models,$IN=0$, since then the expectation surface is planar (or a straight line when$p=1$).

The expectation surface given in the right panel of Fig. 7 is also for these data but corresponds to use of the reciprocal model function parameterization, ${\eta }_{2}\left(x, \gamma \right)={e}^{-x/\gamma }$, instead of the original model function parameterization, $\eta \left(x, \theta \right)={e}^{-\theta x}$. For ${\eta }_{2}\left(x, \gamma \right)$, the intrinsic curvature is again equal to $IN=1.44$; this follows due to the invariance of $IN$ to the one-to-one reparameterization, $\gamma =1/\theta$.

The data point $({y}_{1},{y}_{2})=(\mathrm{0.93,0.025})$ is also displayed in both panels of Fig. 7. The least-squares estimate points, $\widehat{\theta }=0.5013$ in the left panel and $\widehat{\gamma }=1.9948$ in the right panel, correspond to the point on **E** nearest to the data point.

The second curvature measure, called parameter-effect ($PE$) curvature, assesses the extent to which the spacing of the parameter points on **E** near the LSE point are “regular” (i.e., equi-spaced in this one-parameter case). The spacing in the left panel is distorted since although the actual numerical distance between the numbers $1$ and $0.5013$ is about the same as that between the numbers $0.5013$ to $0$ (i.e., both are about 0.5), the distances between the respective labelled points in the left panel of Fig. 7 are quite different. That is, the arc length of the segment of **E** from the $\theta =1$ point to $\widehat{\theta }=0.50$ is much less than the arc length of the segment of **E** from $\widehat{\theta }=0.50$ to $\theta =0$. Since the $PE$ measure evaluates aspects of spacing, it is not surprising, then, that the left panel’s $PE$ value, $PE=2.43$, greatly exceeds the threshold suggested cut-off value of $0.30$. The $PE$ curvature situation for the right panel in Fig. 7 in the neighborhood of $\widehat{\gamma }=1.99$ is not as problematic since the spacing near $\widehat{\gamma }$ appears more regular. It is not surprising, then, that with the value of $PE=1.03$, although still above the $0.30$ cut-off, the right panel’s parameter-effects curvature measure is less than half that of the left panel. Clearly, for this model and data, both the $IN$ and $PE$ curvature measures are high, and so the Wald approximation will be poor, as was noted above. In practice, note that these curvature values can be reduced by increasing the sample size. We next delve deeper into understanding likelihood and Wald confidence interval methods and any discrepancies between the two.

Using the given data and the original model function, $\eta \left(x, \theta \right)={e}^{-\theta x}$, Fig. 8 enables the visualization of discrepancies between Wald and likelihood confidence intervals. The 80% confidence circle centered at the data point (labelled point $Y$) is obtained from Eq. (5). Instead of plotting this equation in the one-dimensional parameter space, it is viewed here in the $n=2$-dimensional sample space and so it is the (circular) set of $({\eta }_{1},{\eta }_{2})$ values for which

38 $${\left(0.93-{\eta }_{1}\right)}^{2}+{\left(0.025-{\eta }_{2}\right)}^{2}=S\left(\widehat{\theta }\right)\left(1+{t}_{0.10, 1}^{2}\right)=0.3669={0.6057}^{2}$$

The tangent line to **E** at the point $\widehat{\theta }=0.5013$ is also plotted in Fig. 8. It has slope ${\left.\frac{\partial ({\eta }_{1}^{8})}{\partial {\eta }_{1}}\right|}_{{\widehat{\eta }}_{1}=0.78}=1.38$ and is given by the equation ${\eta }_{2}=-0.94+1.38 {\eta }_{1}$. To facilitate understanding of parameter effects curvature, superimposed on this tangent line in Fig. 8 are a series of points (labelled in the plot as filled squares) which have regularly-spaced $\theta$ values; these values are chosen here to be $\theta =-0.25, 0, 0.25,\dots , 1, 1.25$.

As noted above, the intrinsic curvature measure ($IN$) assesses the discrepancy between the actual expectation surface **E** and the tangent line approximation. In this case, this discrepancy is pronounced and is reflected in the fact that the observed $IN=1.44$ exceeds the 0.30 cut-off. The parameter effects curvature measure ($PE$), on the other hand, measures the difference between the actual spacing of the $\theta$ values on **E** (see the left panel of Fig. 7) and the filled-square regularly-spaced $\theta$ values superimposed on the tangent line in Fig. 8. This difference is also pronounced and is reflected in the calculated value, $PE=2.43$, also exceeding 0.30. (In models with more than one model function parameter, $PE$ assesses the degree to which ${\varvec{\theta}}$ values on **E** near $\widehat{{\varvec{\theta}}}$ behave like a regular grid.)

Figure 8 shows that the intersection of the 80% confidence circle given in Eq. (38) with the expectation surface **E** occurs at point A (for which ${\eta }_{1}=0.94$ and ${\eta }_{2}=0.63$) and point B (for which ${\eta }_{1}=0.32$ and ${\eta }_{2}=0.0001$). Using the model function $\eta ={e}^{-\theta x}$ and solving for the corresponding values of $\theta$, these points give the 80% LBCI, $(0.12, 2.25)$. The intersection of the 80% confidence circle with the tangent line approximation occurs at point C (for which ${\eta }_{1}=1.12$ and ${\eta }_{2}=0.60$) and point D (for which ${\eta }_{1}=0.44$ and ${\eta }_{2}=-0.33$). Using the linear approximation to the model function, $\eta (x,\theta )\cong {e}^{-\widehat{\theta }x}-x{e}^{-\widehat{\theta }x}(\theta -\widehat{\theta })$ and solving for $\theta$, these points give the 80% WCI, $(-0.37, 1.37)$. This demonstrates how Wald and likelihood confidence intervals are obtained for nonlinear models and how and why they differ.

This simple, one-parameter, small ($n=2$) illustration demonstrates that Wald confidence intervals are obtained based on two approximations, which may or may not be met for a given nonlinear model and dataset. The first approximation involves replacing the actual expectation surface with its tangent line (or tangent plane or hyper-plane) approximation. The second approximation involves replacing the actual spacing of the parameter values on the expectation surface near the parameter estimate with a regular grid, and using this approximate regular grid to measure distances. For the reciprocal parameterization used above for this model (and with expectation surface in the right panel of Fig. 7), the spacing of the parameter values on the expectation surface in the vicinity of $\widehat{\gamma }$ is more regular, and so the $PE$ curvature would therefore be lower. Since commonly-used statistical software typically does not indicate when these approximations hold or fail, practitioners are wise to bear them in mind when using (approximate) Wald confidence intervals and p-values. ■

For the laetisaric illustration in Example 2 (Sect. 2) and the Fieller-Creasy illustration in Example 5 (Sect. 4), the intrinsic curvatures measures are exactly zero. This follows since the chosen model functions are transformations of linear models, the $IN$ measure is invariant to the reparameterization of the model function, and $IN$ is zero for linear models. For example, Example 2’s model function, $\eta \left(x,{\varvec{\theta}}\right)=\alpha \left(1-\frac{x}{2\theta }\right)$, is a one-to-one transformation of the usual linear regression model function, $\eta \left(x,{\varvec{\theta}}\right)=\alpha +\beta x$, using the non-singular transformation from $(\alpha ,\beta )$ to $(\alpha ,\theta )$ involving $\theta =-\frac{\alpha }{2\beta }$. This means that differences between PLCIs and WCIs for these examples result only from parameter-effects curvature, and that PLCIs are exact for these illustrations.

In addition to the two overarching reasons given in Sect. 3.4 for preferring likelihood intervals over Wald intervals, a third reason is related to model reparameterization: likelihood intervals are invariant to reparameterizations—even nonlinear ones—but Wald intervals are not. To illustrate, recall that the model function used in Example 3 is${\eta }_{1}\left(x,\theta \right)=\frac{x}{\theta +x}$, where the model parameter ($\theta$) is the${EC}_{50}$. The LSE is $\widehat{\theta }=5.8698$ and the likelihood and Wald confidence intervals for $\theta$ are $\left(2.9960 , 12.7122\right)$ and $\left(1.8106 , 9.9291\right)$ respectively. To assess overlap in these intervals, the intersection of these intervals is the interval, $\left(2.9960 , 9.9291\right)$ and the union of these intervals is the interval,$\left(1.8106 , 12.7122\right)$. These latter intervals have respective lengths 6.9331 and 10.9016 and an assessment of overlap of the LBCI and WCI (Haines et al. 2004) is$\frac{6.9331}{10.9016}=63.60\%$. This shows a fair amount of difference between these two intervals. A simple modification of the original model function is${\eta }_{2}\left(x,\phi \right)=\frac{\phi x}{1+\phi x}$, so that this new model function’s parameter ($\phi$) is the reciprocal ${EC}_{50}$ since when $\phi =1/\theta$ is substituted here, we obtain the original model function. When this new modified model function is fit to the data, we obtain $\widehat{\phi }=0.1704$ (which equals$1/\widehat{\theta }$) and the respective likelihood and Wald confidence intervals for $\phi$ are $\left(0.07867 , 0.3338\right)$ and$\left(0.05255 , 0.2882\right)$. Notice first that (except for roundoff error) the reciprocal of the ${\eta }_{2}\left(x,\phi \right)$ LBCI endpoints, $\frac{1}{0.07867}=12.7113$ and$\frac{1}{0.3338}=2.9960$, coincide exactly with the LBCI endpoints of ${\eta }_{1}\left(x,\theta \right)$ given above. This invariance does not hold for WCI’s, since $\frac{1}{0.05255}=19.029\ne 9.9291$ and$\frac{1}{0.2882}=3.4698\ne 1.8106$. Further, for these data and${\eta }_{2}\left(x,\phi \right)$, the overlap assessment of the LBCI and WCI is$\frac{0.2095}{0.2812}=74.50\%$; since this value exceeds the $63.60\%$ overlap assessment for the original parameterization, there is more agreement here between the LBCI and WCI. Thus, in general and also in multidimensional ($p>1$) situations, likelihood intervals are invariant to one-to-one parameter modifications and some reparameterizations yield closer agreement between likelihood and Wald intervals than others.

## Appendix A.2. Distinguishing Two Likelihood Approaches and Penalizing for Overfitting

An internet search of the term, “profile likelihood confidence interval,” yields several references to asymptotic likelihood tests and intervals (Royston 2007; Stryhn and Christensen 2003; Venzon and Moolgavkar 1988). The intentional focus in our work has been solely on model function parameters in homoscedastic normal nonlinear models which use the F-based (exact or nearly-exact) likelihood-based expressions. Along these lines, we next distinguish the two different likelihood approaches and discuss penalizing for overfitting related to profiled-out model function parameters.

In contrast with the F-statistic likelihood approach used in this paper, the approximate (or asymptotic) likelihood-based test for testing ${H}_{0}:{\varvec{\theta}}={{\varvec{\theta}}}_{0}$, is based on twice the change in the log-likelihood (denoted $LL$),

39 $$2\left(LL\left({\widehat{{\varvec{\theta}}}}_{MLE}\right)-LL\left({{\varvec{\theta}}}_{0}\right)\right)$$

(Recall that the log-likelihood for the normal distribution is given in Eq. (4).) In Eq. (39), ${\widehat{{\varvec{\theta}}}}_{MLE}$ maximizes $LL({\varvec{\theta}})$. In very general situations, this asymptotic (i.e., valid for large sample sizes) test statistic approximately follows the chi-square distribution with $p$ degrees of freedom. In a manner similar to Eq. (16), this test statistic can be “inverted” to obtain approximate likelihood intervals (see Eq. (40) below). The asymptotic likelihood approach is commonly used in generalized linear, survival and longitudinal modelling. For the homoscedastic normal cases considered here, these large-sample likelihood intervals and tests generally differ from the F-statistics likelihood methods, and F-statistic likelihood methods are preferred for the reasons given later in this section (and also due to increased power).

When comparing the F-based likelihood approach used in this work and the approximate likelihood approach in Eq. (39), in addition to better coverage, another important reason for preferring the F-based likelihood approach is that they levy a penalty for overfitting, and we demonstrate this as follows. Using model parameter profiling and inverting Eq. (39), the approximate profile likelihood confidence interval (APLCI) for key parameter ${\theta }_{2}$ is the set of ${\theta }_{2}$ for which

40 $$LL\left({\widetilde{{\varvec{\theta}}}}_{1},{\theta }_{2}\right)=LL\left(\widehat{{\varvec{\theta}}}\right)-\frac{1}{2}{\chi }_{\alpha ,1}^{2}$$

Here, ${\chi }_{\alpha ,1}^{2}$ is the $(1-\alpha )100\%$ quantile of the distribution with one degree of freedom and is such that $P\left({\chi }^{2}>{\chi }_{\alpha ,1}^{2}\right)=\alpha$. On the other hand, as stated in the main text and repeated here, the (exact or nearly exact) F-statistic-based profile likelihood confidence interval (PLCI) is the set of ${\theta }_{2}$ values which solve the equation

41 $$S({\widetilde{{\varvec{\theta}}}}_{1},{\theta }_{2})=S\left(\widehat{{\varvec{\theta}}}\right)\left(1+\frac{{t}_{\left(\alpha /2\right),(n-p)}^{2}}{n-p}\right)$$

Since the APLCI approach is based on the chi-square quantile with one degree of freedom, it treats the single-parameter situation and the multi-parameter situation the same. That is, in the multiparameter case, the approximate approach profiles out the nuisance parameter(s) and treats the resulting profile equation as a one-parameter likelihood equation, so it levies no penalty for estimating the profiled parameters. The preferred PLCI approach of Eq. (41), on the other hand, is based on the $t$ distribution with $(n-p)$ degrees of freedom, which penalizes for the estimation of the $(p-1)$ other (i.e., nuisance) parameters. Indeed, this PLCI statistic calibrates the result since for fixed $n$ and $\alpha$, the term

42 $$\frac{{t}_{\left(\alpha /2\right),(n-p)}^{2}}{n-p}$$

increases with the number of parameters, $p$. This means that for a larger total number of nonlinear model parameter to estimate, the cut-line of the profile function will be higher, and the resulting profile confidence interval will be wider (i.e., more conservative), thereby reflecting the penalty for estimating a larger number of parameters. The interested reader can visualize these results by examining Fig. 2.

Other works (Fears et al. 1996; Pawitan 2000) have highlighted Wald confidence interval anomalies however their examples have been less clear-cut and have been based on the approximate likelihood test. Focusing on variance component estimation in the one-way random effects analysis of variance (ANOVA) model, these works underscore the inadequacy of Wald method using both simulation and this approximate or large-sample likelihood approach. Estimation of the variance component in this one-way random-effects ANOVA case is problematic since the null hypothesis value (i.e., the zero in the expression, ${H}_{0}:{\sigma }_{0}^{2}=0$) is on the boundary of the parameter space and so the likelihood distribution statistic has a mixed distribution (Chernoff 1954; Molenberghs and Verbeke 2007). As such, although the random-effects ANOVA illustration does underscore Wald inadequacies, it easily confuses practitioners who may confound boundary issues with Wald statistics caveats and so may not realize the far-reaching nature of these Wald inadequacies. Our chosen nonlinear regression examples, on the other hand, underscore some nuances associated with nonlinear modelling and clearly show the extent of the preference of F-based likelihood methods over Wald methods.

In sum, with strong evidence in favor of likelihood methods over Wald methods—and the F-statistic-based likelihood methods considered here over the APLCI likelihood approach in Eq. (40)—it is surprising that some software packages still report Wald (and sometimes approximate likelihood) p-values and intervals for homoskedastic normal nonlinear models.

## Appendix A.3. Some Detailed Results Related to the Fieller-Creasy Problem

For the Fieller-Creasy model function given in Eq. (29), the sum-of-squares function is

43 $$S\left({\varvec{\theta}}\right)={\sum }_{j=1}^{{n}_{1}}{\left({y}_{1j}-{\theta }_{1}\right)}^{2}+{\sum }_{j=1}^{{n}_{2}}{\left({y}_{2j}-{\theta }_{1}{\theta }_{2}\right)}^{2}$$

Differentiating in Eq. (43) with respect to the model parameters and setting to zero, the LSE parameter estimates are ${\widehat{\theta }}_{1}={\overline{y} }_{1}$ and ${\widehat{\theta }}_{2}={\overline{y} }_{2}/{\overline{y} }_{1}$ and the residual sum-of-squares is $S\left(\widehat{{\varvec{\theta}}}\right)={\sum }_{j=1}^{{n}_{1}}{\left({y}_{1j}-{\overline{y} }_{1}\right)}^{2}+{\sum }_{j=1}^{{n}_{2}}{\left({y}_{2j}-{\overline{y} }_{2}\right)}^{2}$. We restrict attention here to situations where ${\theta }_{1}$, ${\theta }_{2}$, ${\overline{y} }_{1}$ and ${\overline{y} }_{2}$ are all positive since absolute value terms can be substituted otherwise. The $n\times 2$ Jacobian matrix ${\varvec{X}}$ has the first ${n}_{1}$ rows equal to one in column one and to zero in column two, and the last ${n}_{2}$ rows equal to ${\theta }_{2}$ in column one and to ${\theta }_{1}$ in column two. It follows that

44 $${{\varvec{X}}}^{T}{\varvec{X}}=\left[\begin{array}{cc}{n}_{1}+{n}_{2}{\theta }_{2}^{2}& {n}_{2}{\theta }_{1}{\theta }_{2}\\ {n}_{2}{\theta }_{1}{\theta }_{2}& {n}_{2}{\theta }_{1}^{2}\end{array}\right]$$

For this multi-parameter ($p=2$) situation, the likelihood-based confidence region is the set of θ such that

45 $$S({\varvec{\theta}})=S\left(\widehat{{\varvec{\theta}}}\right)\left(1+{\frac{p}{n-p}F_{\alpha ,p,(n-p)}}\right)$$

In this instance and $\alpha =0.05$, the right-hand-side of this expression is $S\left(\widehat{{\varvec{\theta}}}\right)\left(1+{\frac{p}{n-p}F}_{\alpha ,p,(n-p)}\right)=20\left(1+\frac{2}{9}\times 4.2565\right)=38.92$. Further, the (Wald) approximation to $S\left({\varvec{\theta}}\right)-S\left(\widehat{{\varvec{\theta}}}\right)$ here is

46 $$\left({n}_{1}+{n}_{2}{\widehat{\theta }}_{2}^{2}\right){\left({\theta }_{1}-{\widehat{\theta }}_{1}\right)}^{2}+{2{n}_{2}\widehat{\theta }}_{1}{\widehat{\theta }}_{2}\left({\theta }_{1}-{\widehat{\theta }}_{1}\right)\left({\theta }_{2}-{\widehat{\theta }}_{2}\right)+{n}_{2}{\widehat{\theta }}_{1}^{2}{\left({\theta }_{2}-{\widehat{\theta }}_{2}\right)}^{2}$$

For these data, the 95% likelihood confidence region (solid region) and the 95% Wald confidence region (dashed ellipse) are plotted in the left panel of Fig. 9. The plotted central point in the figure is the least-squares estimator, ${\widehat{{\varvec{\theta}}}}^{T}=\left(4, 2\right)$. These regions are ‘joint’ or ‘simultaneous’ confidence regions for the parameter vector ${{\varvec{\theta}}}^{T}=\left({\theta }_{1},{\theta }_{2}\right)$, and in a similar manner to the confidence *interval* case, simulation results (Donaldson and Schnabel 1987; Seber and Wild 1989) highlight the superiority of likelihood *regions* over Wald *regions* in the sense of better agreement between nominal and actual coverage probabilities (e.g., 95%).

Another manner to obtain the Wald approximate region in Eq. (46) is to observe that for this model function the first-order (planar) Taylor approximation of $\eta \left(x,{\varvec{\theta}}\right)$ about the point (${\widehat{\theta }}_{1},{\widehat{\theta }}_{2}$) is

47 $$\eta \left(x,{\varvec{\theta}}\right)=\left\{\begin{array}{c}{\theta }_{1} \\ {\theta }_{1}{\theta }_{2}\end{array}\cong \left\{\begin{array}{c}{\widehat{\theta }}_{1}+\left({\theta }_{1}-{\widehat{\theta }}_{1}\right) \quad (group 1)\\ {\widehat{\theta }}_{1}{\widehat{\theta }}_{2}+{\widehat{\theta }}_{2}\left({\theta }_{1}-{\widehat{\theta }}_{1}\right)+{\widehat{\theta }}_{1}\left({\theta }_{2}-{\widehat{\theta }}_{2}\right) \quad (group 2)\end{array}\right.\right.$$

Substituting these approximations into the sum of squares expression in Eqs. (5) and (45) gives the Wald confidence region expression given in Eq. (46).

To obtain the PLCI for ${\theta }_{2}$ we profile out ${\theta }_{1}$ by setting to zero the corresponding normal equation,

48 $${\sum }_{j=1}^{{n}_{1}}\left({y}_{1j}-{\theta }_{1}\right)+{\theta }_{2}{\sum }_{j=1}^{{n}_{2}}\left({y}_{2j}-{\theta }_{1}{\theta }_{2}\right)=0$$

Next, when ${\theta }_{2}$ is taken as fixed and this expression is solved for ${\theta }_{1}$, we obtain the constrained optimum value,

49 $${\widetilde{\theta }}_{1}=\frac{{n}_{1}{\widehat{\theta }}_{1}+{n}_{2}{\widehat{\theta }}_{1}{\widehat{\theta }}_{2}{\theta }_{2}}{{n}_{1}+{n}_{2}{\theta }_{2}^{2}}=\frac{{n}_{1}}{{n}_{1}+{n}_{2}{\theta }_{2}^{2}}{\widehat{\theta }}_{1}+\frac{{n}_{2}{\theta }_{2}^{2}}{{n}_{1}+{n}_{2}{\theta }_{2}^{2}}\frac{{\widehat{\theta }}_{2}}{{\theta }_{2}}{\widehat{\theta }}_{1}$$

Interestingly, Eq. (49) is of the form $\omega {\widehat{\theta }}_{1}+(1-\omega )\frac{{\widehat{\theta }}_{2}}{{\theta }_{2}}{\widehat{\theta }}_{1}$, which is a weighted sum of the LSE, ${\widehat{\theta }}_{1}$, and the modified estimator $\frac{{\widehat{\theta }}_{2}}{{\theta }_{2}}{\widehat{\theta }}_{1}$ with $\omega =$ $\frac{{n}_{1}}{{n}_{1}+{n}_{2}{\theta }_{2}^{2}}$. The LSE weight $\omega$ increases as ${n}_{1}$ increases relative to ${n}_{2}$, as ${\theta }_{2}={\mu }_{2}/{\mu }_{1}$ decreases, or as ${\theta }_{2}$ nears ${\widehat{\theta }}_{2}$.

Next, the constrained maximal value ${\widetilde{\theta }}_{1}$ of Eq. (49) is substituted into the profile log-likelihood expression to obtain

50 $$S\left({\widetilde{\theta }}_{1},{\theta }_{2}\right)-S\left(\widehat{{\varvec{\theta}}}\right)=\frac{{n}_{1}{n}_{2}{\widehat{\theta }}_{1}^{2}}{{n}_{1}+{n}_{2}{\theta }_{2}^{2}}{\left({\theta }_{2}-{\widehat{\theta }}_{2}\right)}^{2}$$

The Wald counterpart to the adjusted profile log-likelihood expression in Eq. (50) is very similar but with the right-hand side instead equal to

51 $$\frac{{n}_{1}{n}_{2}{\widehat{\theta }}_{1}^{2}}{{n}_{1}+{n}_{2}{\widehat{\theta }}_{2}^{2}}{\left({\theta }_{2}-{\widehat{\theta }}_{2}\right)}^{2}$$

This subtle but crucial change to Eq. (50)—whereby ${\theta }_{2}^{2}$ in the denominator is replaced by the estimated value ${\widehat{\theta }}_{2}^{2}$—is cause for the difference between WCIs and PLCIs for this model. These details supply the justification of the WCI and PLCI expressions given in Eqs. (30) and (31).

For the given data, these shifted profile expressions are plotted in the right-hand panel of Fig. 9. The profile log-likelihood is the solid curve and the Wald approximation is the dashed parabola. The minimum value point occurs at $({\widehat{\theta }}_{2}, 0)$. The cut-lines in Fig. 9’s right-hand panel occur at ${s}^{2}{t}_{\left(\alpha /2\right),(n-2)}^{2}=(20/9){t}_{\left(\alpha /2\right),9}^{2}$ which is equal to $11.37$ for 95% and to $23.47$ for 99%. For the given data, the 95% PLCI, $(1.30, 3.94)$, are points ${A}_{95}$ and ${B}_{95}$ in the right panel of Fig. 9, and the 95% WCI, $(0.98, 3.02)$, are points ${C}_{95}$ and ${D}_{95}$. Thus, for these data, the Wald interval contains the value of one and so would retain the hypothesis of equal means (${H}_{0}:{\theta }_{2}=1$). But since the PLCI does not contain one, the equal means hypothesis would be rejected. Further, the 99% PLCI, $(1.11, 6.71)$ (points ${A}_{99}$ to ${B}_{99}$ in the graph), represent a large right shift from the 99% WCI, $(0.54, 3.46)$ (points ${C}_{99}$ to ${D}_{99}$ in the graph). Shifts in the center of PLCIs (versus WCIs) as well as lengthening or shortening of these intervals are related to skewness and curvature measures (Clarke 1987; Haines et al. 2004; Ratkowsky 1983).

It is noteworthy to underscore the following regarding model reparameterization here. Again for $x=1$ for group 1 observations and $x=0$ for group 2 observations, we now rewrite (or reparameterize) the Fieller-Creasy model function in Eq. (29) as

52 $${\eta }_{2}\left(x,{\varvec{\theta}}\right)={\theta }_{1}x+\left({\theta }_{1}/{\theta }_{2}^{R}\right)\left(1-x\right)$$

So ${\eta }_{2}\left(x,{\varvec{\theta}}\right)={\theta }_{1}={\mu }_{1}$ for group 1 (as before) but now ${\eta }_{2}\left(x,{\varvec{\theta}}\right)={\theta }_{1}/{\theta }_{2}^{R}$ for those in group 2. Thus, ${\theta }_{2}^{R}={\mu }_{1}/{\mu }_{2}$ is the reciprocal value of the original ${\theta }_{2}={\mu }_{2}/{\mu }_{1}$. In this reciprocal case, the 95% PLCI for ${\theta }_{2}^{R}$ is $(0.25, 0.77)$, which is precisely the reciprocal of the 95% PLCI for ${\theta }_{2}$, $(1.30, 3.94)$. This again underscores the fact that PLCIs are invariant to one-to-one (even nonlinear) transformations. The 95% WCI for ${\theta }_{2}^{R}$ is $(0.25, 0.75)$, whereas the reciprocal of this WCI, $(1.33, 4.07)$, differs substantially from the original 95% WCI for ${\theta }_{2}$, $(0.98, 3.02)$. This invariance of the PLCI to reparameterization—and lack of invariance of the WCI—reemphasizes the important advantage of likelihood methods over Wald methods. Indeed, the coincidence of the PLCI and WCI on the reciprocal scale (i.e., for ${\theta }_{2}^{R}$) but not on the original scale (i.e., for ${\theta }_{2}$) also highlights the statistical curvature attributes associated with this dataset and these models. We observed similar results in Sect. 3.4 for the single-parameter model function and LBCI used in Example 3, but here these results extend to the $p=2$ case and PLCI situation.
