# Supplementary material for: Nonlinear Regression Modelling: A Primer with Applications and Caveats
Source: Bull Math Biol. 2024 Mar 15;86(4):40. doi: 10.1007/s11538-024-01274-4 (PMC10943168; doi:10.1007/s11538-024-01274-4)
Supplement: Supplementary file 1 — Supplementary file1 (PDF 206 KB) [file 11538_2024_1274_MOESM1_ESM.pdf]

Supplementary Information

Article Title: Nonlinear Regression Modelling: A Primer with Applications and Caveats

Journal Name: Bulletin of Mathematical Biology

Authors: Timothy E. O'Brien[1*] and Jack W Silcox[2]

[1]Department of Mathematics and Statistics, Loyola University Chicago, Chicago, IL, USA; * Corresponding author (Email: tobrie1@luc.edu).

[2]Department of Psychology, University of Utah, Salt Lake City, UT, USA

```
# Figure 1. Michaelis-Menten and Laetisaric Acid Plots
# Examples 1 and 2

  par(mfrow = c(1, 2))
# Left Panel - Michaelis-Menten Enzyme Kinetics
  conc     <- rep(seq(0.02, 0.10, by=0.02), each=3)
  velocity <- c( 58,  28,  58,  78,  65,  87, 104, 110, 109,
                106, 127, 118, 113, 125, 134)
  enzyme   <- data.frame(conc, velocity)
  MM <- function(x,th1,th2) th1*x/(th2+x)
  result <- nls(velocity ~ MM(conc,th1,th2), data=enzyme,
               start=list(th1=250, th2=0.1), trace=F)
  summary(result)
  PE <- coef(result)
  SE <- sqrt(diag(vcov(result)))
  xrange <- range(0, 0.13)
  yrange <- range(0, 150)
  plot(conc, velocity, pch=19,
       xlim=xrange, ylim=yrange,
       xlab="Substrate concentration", ylab="Enzyme velocity")
  xx <- seq(0,0.13,length=1000)
  yy <- MM(xx, PE[1], PE[2])
  par(new=T)
  plot(xx, yy, type="l", lwd=3,
       xlim=xrange, ylim=yrange,
       xlab="", ylab="")
  points(PE[2], 0.5*PE[1], pch=17, cex=1.8, col="darkred")
  yyV <- seq(0, 0.5*PE[1], length=2)
  xxV <- rep(PE[2], 2)
  lines(xxV, yyV, lwd=1, col="black", lty=2)
  yyH <- rep(0.5*PE[1], 2)
  xxH <- seq(0, PE[2], length=2)
  lines(xxH, yyH, lwd=1, col="black", lty=2)
# Right Panel - Laetisaric Acid
  laetConc   <- c( 0,    3,    6,   10,   20,   30)
  fungGrowth <- c(31.2, 33.1, 23.1, 29.7, 18.8, 9.5)
  (fungus <- data.frame(laetConc, fungGrowth))
  plot(laetConc, fungGrowth, cex=1, pch=19,
       xlim=c(0, 32), ylim=c(0, 35),
       xlab="Laetisaric Acid Concentration", ylab="Fungal Growth")
  LaetLine <- function(x, alf, gam) alf*(1 - 0.5*x/gam)
  fit.LL <- nls(fungGrowth ~ LaetLine(laetConc, alf, gam),
               data=fungus, start=list(alf=20, gam=25), trace=F)
  summary(fit.LL)
  conc <- seq(0, 31, length=2)
  pred.LL <- LaetLine(conc, coef(fit.LL)[1], coef(fit.LL)[2])
  lines(conc, pred.LL, lwd=3, col="black")
  alfE <- coef(fit.LL)[1]
```

```
  gamE <- coef(fit.LL)[2]
  y50E <- LaetLine(gamE, alfE, gamE)
  points(gamE, y50E, cex=1.8, col="darkred", pch=17)
  yyV <- seq(0, y50E, length=2)
  xxV <- rep(gamE, 2)
  lines(xxV, yyV, lwd=1, col="black", lty=2)
  yyH <- rep(y50E, 2)
  xxH <- seq(0, gamE, length=2)
  lines(xxH, yyH, lwd=1, col="black", lty=2)
  par(mfrow = c(1,1))

# Figure 2.  Example 3. MM1 and simulated data
  x <- c(0, 2, 4, 6)
  y <- c(0.036910, 0.208884, 0.519404, 0.429751)
  MM1 <- function(x,th) x/(x+th)
  fit <- nls(y ~ MM1(x,theta), start=list(theta=4))
  summary(fit)
  NE <- function(theta) {
    sum <-
      (0.036910 - 0/(0+theta))*(0/(theta+0)^2) +
      (0.208884 - 2/(2+theta))*(2/(theta+2)^2) +
      (0.519404 - 4/(4+theta))*(4/(theta+4)^2) +
      (0.429751 - 6/(6+theta))*(6/(theta+6)^2)
    sum
  }
  soln <- uniroot(NE, c(0.001, 20))
  (thHat <- soln$root)
  Sth <- function(theta) {
    sum <-
      (0.036910-0)^2 +
      (0.208884-2/(2+theta))^2 +
      (0.519404-4/(4+theta))^2 +
      (0.429751-6/(6+theta))^2
    sum
  }
  (Shat  <- Sth(thHat))
  (MSE <- Shat/3)
  (XTX <- 0^2/(thHat+0)^4 + 2^2/(thHat+2)^4 +
      4^2/(thHat+4)^4 + 6^2/(thHat+6)^4)
  (SE <- sqrt(MSE/XTX))
  #LBCI  <- confint(fit)
  LBCIroots <- function(theta) {
    sum <-
      (0.036910-0)^2 +
      (0.208884-2/(2+theta))^2 +
      (0.519404-4/(4+theta))^2 +
      (0.429751-6/(6+theta))^2
    toZero <- sum-Shat*( 1+(qt(0.975,3)^2)/3 )
  }
```

```
  solnL <- uniroot(LBCIroots, c(0.001, 5))
  LBCIL <- solnL$root
  solnR <- uniroot(LBCIroots, c(6, 15))
  LBCIR <- solnR$root
  cbind(LBCIL,LBCIR)
  WCIL   <- coef(fit) - qt(0.975,3)*SE
  WCIR   <- coef(fit) + qt(0.975,3)*SE
  cbind(WCIL, WCIR)
# Create Figure 2
# This is the LEFT PANEL - i.e, for MM1 Model
  par(mfrow=c(1,2))
  thLBCI <- seq(2.9, 13, length=1000)
  yyLBCI <- Sth(thLBCI)
  thWCI  <- seq(1.75, 10, length=1000)
  th     <- seq(1.75, 13, length=1000)
  yyWCI  <- Sth(thHat) + 0.004542415*(thWCI - thHat)^2
  yrange <- range(yyWCI,yyLBCI)
  xrange <- range(thLBCI, thWCI)
  (ycut <- 0.0221707*(1+(1/3)*qt(0.975,3)^2))
    0.0221707*(1+(1/3)*qt(0.95,3)^2)  # aside cut for 90%
  sqrt((ycut-Sth(thHat))/0.004542415)
  qt(0.975,3)*1.27552
  yFlat  <- rep(ycut, 1000)
  plot(thLBCI, yyLBCI, type="l", lwd=3, ylim=yrange, xlim=xrange,
       xlab=expression(paste(theta, ": EC50 parameter")),
       ylab=expression(paste("S(",theta, "): Sum of Squares")))
  par(new=T)
  plot(th, yFlat, type="l", lwd=2, lty=3, ylim=yrange, xlim=xrange,
       xlab="", ylab="")
  points(thHat, Shat, pch=19, cex=2)
  points(LBCIL, ycut, pch=15, cex=1.5)
  points(LBCIR, ycut, pch=15, cex=1.5)
  par(new=T)
  plot(thWCI, yyWCI, type="l", lwd=3, lty=2, ylim=yrange, xlim=xrange,
       xlab="", ylab="")
  points(WCIL, ycut, pch=17, cex=1.5)
  points(WCIR, ycut, pch=17, cex=1.5)
  # End make Figure 2 LEFT PANEL
  Sth(2.99602)
  Sth(12.7122)
# Figure 2 RIGHT PANEL: Example 2 reprise
  laetConc   <- c(0, 3, 6, 10, 20, 30)
  fungGrowth <- c(31.2, 33.1, 23.1, 29.7, 18.8, 9.5)
  xx <- laetConc
  yy <- fungGrowth
  PsseF <- function(gam) {
    dif1 <- 1 - 0.5*xx[1]/gam
    dif2 <- 1 - 0.5*xx[2]/gam
    dif3 <- 1 - 0.5*xx[3]/gam
```

```
  dif4 <- 1 - 0.5*xx[4]/gam
  dif5 <- 1 - 0.5*xx[5]/gam
  dif6 <- 1 - 0.5*xx[6]/gam
  top <- yy[1]*dif1 + yy[2]*dif2 + yy[3]*dif3 +
    yy[4]*dif4 + yy[5]*dif5 + yy[6]*dif6
  bot <- dif1*dif1 + dif2*dif2 + dif3*dif3 +
    dif4*dif4 + dif5*dif5 + dif6*dif6
  alft <- top/bot
  (yy[1] - alft*dif1)^2 + (yy[2] - alft*dif2)^2 +
    (yy[3] - alft*dif3)^2 + (yy[4] - alft*dif4)^2 +
    (yy[5] - alft*dif5)^2 + (yy[6] - alft*dif6)^2 -
    56.845121514 - (56.845121514/4)*qf(0.95,1,4)
}
solnL <- uniroot(PsseF, c(10, 22))
LBCIL <- solnL$root
solnR <- uniroot(PsseF, c(22, 60))
LBCIR <- solnR$root
cbind(LBCIL, LBCIR)
PsseF(22.3271)
PsseF(15.91761)
PsseF(43.96402)
gamp <- seq(15.6, 47.5, length=1000)
Profsse <- PsseF(gamp)
xrange <- range(12.5, 47.5)
yrange <- range(Profsse)
plot(gamp, Profsse, type="l", lwd=3, ylim=yrange, xlim=xrange,
     ylab="Shifted Profiled SSE",
     xlab=expression(paste(gamma, ": EC50 parameter")))
points(22.3271, PsseF(22.3271), cex=2, pch=19)
#title("Profiled/Constrained SSE for x50: Laetisaric Acid")
soln <- uniroot(PsseF, c(15, 20))
(left <- soln$root)
soln <- uniroot(PsseF, c(35, 55))
(right <- soln$root)
# PLCI
points( left, 0, cex=1.5, pch=15)
points(right, 0, cex=1.5, pch=15)
xflat <- seq(12.5, 47.5, length=1000)
yflat <- rep(0, 1000)
par(new=T)
plot(xflat, yflat, type="l", lwd=2, lty=3,
     ylim=yrange, xlim=xrange,
     ylab="", xlab="")
# Find PLCI of 15.91761 to 43.96402 matches above NLP result.
# Add Wald parabolic approximation to profile curve
thWCI  <- seq(12.1434, 32.5108, length=1000)
yyWCI  <- ((thWCI - 22.3271)^2)/0.8674898 - 109.5498
par(new=T)
plot(thWCI, yyWCI, type="l", lwd=3, lty=2,
```

```
      ylim=yrange, xlim=xrange, ylab="", xlab="")
  points(12.57899, 0, cex=1.5, pch=17)
  points(32.07518, 0, cex=1.5, pch=17)
  par(mfrow=c(1,1))
# Reparameterizing MM1 to check for invariance of CIs
  x <- c(0, 2, 4, 6)
  y <- c(0.036910, 0.208884, 0.519404, 0.429751)
  MM1b <- function(x,phi) phi*x/(1+phi*x)
  fit <- nls(y ~ MM1b(x,phi), start=list(phi=0.3))
  summary(fit)
  NE <- function(phi) {
    sum <-
      (0.036910 - 0*phi/(1+0*phi))*(0/(1+0*phi)^2) +
      (0.208884 - 2*phi/(1+2*phi))*(2/(1+2*phi)^2) +
      (0.519404 - 4*phi/(1+4*phi))*(4/(1+4*phi)^2) +
      (0.429751 - 6*phi/(1+6*phi))*(6/(1+6*phi)^2)
    sum
  }
  soln <- uniroot(NE, c(0.00001, 1))
  (phiHat <- soln$root)
  Sphi <- function(phi) {
    sum <-
      (0.036910-0)^2 +
      (0.208884-2*phi/(1+2*phi))^2 +
      (0.519404-4*phi/(1+4*phi))^2 +
      (0.429751-6*phi/(1+6*phi))^2
    sum
  }
  (Shat  <- Sphi(phiHat))
  (MSE <- Shat/3)
  (XTX <- 0^2/(1+phiHat*0)^4 + 2^2/(1+phiHat*2)^4 +
          4^2/(1+phiHat*4)^4 + 6^2/(1+phiHat*6)^4)
  (SE <- sqrt(MSE/XTX))
  LBCIroots <- function(phi) {
    sum <-
      (0.036910-0)^2 +
      (0.208884-2*phi/(1+2*phi))^2 +
      (0.519404-4*phi/(1+4*phi))^2 +
      (0.429751-6*phi/(1+6*phi))^2
    toZero <- sum-Shat*( 1+(qt(0.975,3)^2)/3 )
  }
  solnL <- uniroot(LBCIroots, c(0.00001, 0.17))
  LBCIL <- solnL$root
  solnR <- uniroot(LBCIroots, c(0.17, 1))
  LBCIR <- solnR$root
  cbind(LBCIL,LBCIR)
  WCIL   <- coef(fit) - qt(0.975,3)*SE
  WCIR   <- coef(fit) + qt(0.975,3)*SE
  cbind(WCIL, WCIR)
```

```
# Figure 3. Michaelis-Menten and Laetisaric Acid Plots NOW WITH CIs
  par(mfrow=c(1,2))
# Left Panel - Michaelis-Menten Enzyme Kinetics
  conc     <- rep(seq(0.02, 0.10, by=0.02), each=3)
  velocity <- c( 58,  28,  58,  78,  65,  87, 104, 110, 109,
                106, 127, 118, 113, 125, 134)
  enzyme   <- data.frame(conc, velocity)
  MM2 <- function(x,th1,th2) th1*x/(th2+x)
  result <- nls(velocity ~ MM2(conc,th1,th2), data=enzyme,
               start=list(th1=250, th2=0.1), trace=F)
  summary(result)
  PE <- coef(result)
  SE <- sqrt(diag(vcov(result)))
  xrange <- range(0, 0.13)
  yrange <- range(0, 150)
  plot(conc, velocity, pch=19,
       xlim=xrange, ylim=yrange,
       xlab="Substrate concentration", ylab="Enzyme velocity")
  xx <- seq(0,0.13,length=1000)
  yy <- MM2(xx, PE[1], PE[2])
  par(new=T)
  plot(xx, yy, type="l", lwd=3,
       xlim=xrange, ylim=yrange,
       xlab="", ylab="")
  points(PE[2], 0.5*PE[1], pch=17, cex=1.8, col="darkred")
  (WCI.th1 <- PE[1] + c(-1,1)*qt(0.975,10)*SE[1])
  (PLCI.th1 <- confint(result)[1,])
  (WCI.th2 <- PE[2] + c(-1,1)*qt(0.975,10)*SE[2])
  (PLCI.th2 <- confint(result)[2,])
  xxW <- seq(WCI.th2[1], WCI.th2[2], length=2)
  yyW <- rep(20, 2)
  xxP <- seq(PLCI.th2[1], PLCI.th2[2], length=2)
  yyP <- rep(7, 2)
  par(new=T)
  plot(xxW, yyW, type="l", lwd=3, lty=3, col="blue",
       xlim=xrange, ylim=yrange,
       xlab="", ylab="")
  points(WCI.th2, c(20,20), cex=1.8, pch=15, col="blue")
  par(new=T)
  plot(xxP, yyP, type="l", lwd=3, lty=5, col="purple",
       xlim=xrange, ylim=yrange,
       xlab="", ylab="")
  points(c(PLCI.th2[1], PLCI.th2[2]), rep(6,2),
         cex=1.8, pch=19, col="purple")
  legend("right",
         legend = c("95% WCI", "95% PLCI"),
         lwd=3, lty=c(3, 5), col=c("blue", "purple"))
# Right Panel - Laetisaric Acid
```

```
  laetConc   <- c( 0,    3,    6,   10,   20,  30)
  fungGrowth <- c(31.2, 33.1, 23.1, 29.7, 18.8, 9.5)
  fungus <- data.frame(laetConc, fungGrowth)
  plot(laetConc, fungGrowth, cex=1, pch=19,
       xlim=c(0, 45), ylim=c(0, 35),
       xlab="Laetisaric Acid Concentration", ylab="Fungal Growth")
  LaetLine <- function(x, alf, gam) alf*(1 - 0.5*x/gam)
  fit.LL <- nls(fungGrowth ~ LaetLine(laetConc, alf, gam),
                data=fungus, start=list(alf=20, gam=25), trace=F)
  summary(fit.LL)
  conc <- seq(0, 31, length=2)
  pred.LL <- LaetLine(conc, coef(fit.LL)[1], coef(fit.LL)[2])
  lines(conc, pred.LL, lwd=3, col="black")
  alfE <- coef(fit.LL)[1]
  gamE <- coef(fit.LL)[2]
  y50E <- LaetLine(gamE, alfE, gamE)
  points(gamE, y50E, cex=1.8, col="darkred", pch=17)
  SE   <- sqrt(vcov(fit.LL)[2,2])
  WALD <- gamE + c(-1,1)*qt(0.975,4)*SE
  waldL <- WALD[1]; waldR <- WALD[2]
  plciL <- 15.917754; plciR <- 43.964118
  xxP <- seq(plciL, plciR, length=2)
  xxW <- seq(waldL, waldR, length=2)
  yyP <- rep(3, 2)
  yyW <- rep(6, 2)
  #title("Fungal Growth with 95% WCI and PLCI")
  lines(xxW, yyW, lwd=3, lty=3, col="blue")
  points(c(waldL, waldR), rep(6, 2), cex=1.8, pch=15, col="blue")
  lines(xxP, yyP, lwd=3, lty = 5, col="purple")
  points(c(plciL, plciR), rep(3, 2), cex=1.8, pch=19, col="purple")
  legend("topright",
         legend = c("95% WCI", "95% PLCI"),
         lwd = 3, lty = c(3, 5), col = c("blue", "purple"))
  par(mfrow = c(1,1))

# Figure 4 Broken Stick Fit to Corn Yield Data: permitted to use these data
  par(mfrow = c(1, 1))
  nitrogen  <- c( 0,   67,   134,   201,   268,   335)
  cornYield <- c(60.9, 75.9,  83.7,  84.3,  81.8,  84.5)
  Knoxville1964 <- data.frame(nitrogen, cornYield)
  xrange <- range(0, 350)
  yrange <- range(55, 90)
  plot(Knoxville1964$nitrogen, Knoxville1964$cornYield,
       pch=19, cex=1.4, col="darkblue",
       xlab="Nitrogen in kg/ha", ylab="Corn Yield in q/ha",
       xlim=xrange, ylim=yrange,
       main="Anderson & Nelson's Knoxville 1964 Corn Yields")
  nit <- seq(0, 350, length = 1000)
  lineFlat <- function(x, alf, bet, kap) {
```

```
    meanL <- alf + bet*x
    meanR <- alf + bet*kap
    mean <- ifelse(x < kap, meanL, meanR)
    mean
  }
  fit.LF <- nls(cornYield ~ lineFlat(nitrogen, alf, bet, kap),
                data = Knoxville1964,
                start = list(alf=50, bet=0.5, kap=90), trace=F)
  pred.LF <- lineFlat(nit, coef(fit.LF)[1], coef(fit.LF)[2],
                      coef(fit.LF)[3])
  plot(Knoxville1964$nitrogen, Knoxville1964$cornYield,
       pch=19, cex=1.5, col="black",
       xlab="Nitrogen in kg/ha", ylab="Corn Yield in q/ha",
       xlim=xrange, ylim=yrange)
  lines(nit, pred.LF, lwd = 3, col = "black")
  points(coef(fit.LF)[3], lineFlat(coef(fit.LF)[3], coef(fit.LF)[1],
                                   coef(fit.LF)[2], coef(fit.LF)[3]),
         cex = 2, col = "darkred", pch = 15)

# For Example 5 FC, see below Figure 9 at the end.
# Figure 5 MM2 continued.  Treated and Untreated.
# Original data in Ex. 1 was Untreated.
  conc     <- rep(rep(seq(0.02, 0.10, by=0.02), each=3), 2)
  velocity <- c(  58,  28,  58,  78,  65,  87, 104, 110, 109,
                 106, 127, 118, 113, 125, 134,
                  72,  63,  76, 108, 111, 129, 142, 122, 142,
                 153, 157, 124, 171, 147, 147)
  trtdU    <- rep(c(1,0), each=15)
  trtdT    <- 1 - trtdU
  enzyme2  <- data.frame(trtdT, trtdU, conc, velocity)
  MM2group <- function(x, dT, dU, th1T, th2T, th1U, th2U) {
    th1=th1T*dT+th1U*dU
    th2=th2T*dT+th2U*dU
    th1*x/(th2+x)
  }
  resFull <- nls(velocity ~ MM2group(conc,trtdT,trtdU,th1T,th2T,th1U,th2U),
                 data=enzyme2,
                 start=list(th1T=240, th2T=0.05, th1U=250, th2U=0.1))
  options(scipen=999)
  summary(resFull)
  MM <- function(x,th1,th2) th1*x/(th2+x)
  resRed0 <- nls(velocity ~ MM(conc,th1,th2),
                 data=enzyme2,
                 start=list(th1=250, th2=0.1))
  summary(resRed0)
  anova(resRed0, resFull)
  MM2groupR1 <- function(x, dT, dU, th1, th2T, th2U) {
    th2=th2T*dT+th2U*dU
    th1*x/(th2+x)
```

```
  }
  resRed1 <- nls(velocity ~ MM2groupR1(conc,trtdT,trtdU,th1,th2T,th2U),
                 data=enzyme2,
                 start=list(th1=250, th2T=0.1, th2U=0.05))
  summary(resRed1)
  anova(resRed1, resFull)
  MM2groupR2 <- function(x, dT, dU, th1, rho, th2T) {
    th2U=rho*th2T
    th2=th2T*dT+th2U*dU
    th1*x/(th2+x)
  }
  resRed2 <- nls(velocity ~ MM2groupR2(conc,trtdT,trtdU,th1,rho,th2T),
                 data=enzyme2,
                 start=list(th1=250, rho=1, th2T=0.05))
  summary(resRed2)
  confint(resRed2)
  coef(resRed1)
# Plot of averages with 2 fitted parallel MM2 fits
  conc      <- rep(seq(0.02, 0.10, by=0.02), 2)
  trtd      <- rep(1:0, each=5)
  mvelocity <- c( 70.333,116.000,135.333,144.667,155.000,
                  48.000, 76.667,107.667,117.000,124.000)
  xrange <- range(0, 0.13)
  yrange <- range(0, 170)
  plot(conc, mvelocity, pch=17+2*trtd,
       xlim=xrange, ylim=yrange,
       xlab="Substrate concentration",
       ylab="Average enzyme velocity")
  xx  <- seq(0,0.13,length=1000)
  MM <- function(x,th1,th2) th1*x/(th2+x)
  yyT <- MM(xx, coef(resRed1)[1], coef(resRed1)[2])
  yyU <- MM(xx, coef(resRed1)[1], coef(resRed1)[3])
  par(new=T)
  plot(xx, yyT, type="l", lwd=3, lty=3,
       xlim=xrange, ylim=yrange,
       xlab="", ylab="")
  par(new=T)
  plot(xx, yyU, type="l", lwd=3,
       xlim=xrange, ylim=yrange,
       xlab="", ylab="")
  points(coef(resRed1)[2], 0.5*coef(resRed1)[1], pch=19, cex=1.8)
  points(coef(resRed1)[3], 0.5*coef(resRed1)[1], pch=17, cex=1.8)
  # MM(0.13,coef(resRed1)[1],coef(resRed1)[2])
  legend("bottomright", legend = c("Treated", "Untreated"),
         lwd=3, lty=c(3,1), pch=c(19,17), cex=1.5)

# Figure 6 Seefeldt: permission given to use these data
  dose <- c(rep(0, 5),     rep(0.066, 10), rep(0.198, 10),
            rep(0.66, 10), rep(1.98,  10), rep(6.6, 10))
```

```
logdose <- log(dose+0.01)
yield <- c(0.1673, 0.1037, 0.2134, 0.1908, 0.2409,
           0.1720, 0.2253, 0.1666, 0.1131, 0.1802,
           0.1715, 0.1759, 0.2153, 0.2067, 0.2074,
           0.1503, 0.1598, 0.2088, 0.1905, 0.1664,
           0.1934, 0.1680, 0.1556, 0.1579, 0.1488,
           0.1424, 0.1331, 0.1168, 0.1570, 0.0932,
           0.1076, 0.1740, 0.1657, 0.1683, 0.1421,
           0.1036, 0.0836, 0.0693, 0.0663, 0.0973,
           0.0932, 0.1285, 0.1128, 0.1126, 0.0640,
           0.0457, 0.0319, 0.0545, 0.0813, 0.0661,
           0.0549, 0.0616, 0.0768, 0.0626, 0.0690)
logyield <- log(yield)
srtyield <- sqrt(yield)
yield.25 <- yield^0.25
seefeldt <- data.frame(dose, logdose, yield,
                       logyield, srtyield, yield.25)
yrangeLY <- range(logyield)
yrangeY.25 <- range(yield.25)
yrangeSY <- range(srtyield)
yrangeY <- range(yield)
xrangeD <- range(dose)
xrangeLD <- range(logdose)
LL4 <- function(x,th1,th2,th3,th4) {
  tt <- (x/th3)^th4
  th2+(th1-th2)/(1+tt)
}
LogLL4 <- function(x,th1,th2,th3,th4) {
  tt <- (x/th3)^th4
  mean <- th2+(th1-th2)/(1+tt)
  log(mean)
}
LL4fita <- nls(logyield ~ LL4(dose,th1,th2,th3,th4), data=seefeldt,
              start=list(th1=-1, th2=-4, th3=2, th4=2))
summary(LL4fita)
LL4fitb <- nls(logyield ~ LogLL4(dose,th1,th2,th3,th4), data=seefeldt,
               start=list(th1=0.25, th2=0.05, th3=1, th4=1.2))
summary(LL4fitb)
xx <- seq(0, 6.6, length=1000)
# yy1 PEs after using y=YIELD and modelling variance:
yy1 <- LL4(xx,  0.1870, 0.04245, 1.1897, 1.1460)
# yy2 PEs after using y=LOG-YIELD and constant variance:
yy2 <- LL4(xx, -1.7111, -3.0571, 1.9004, 1.3073)
par(mfrow = c(1,2))
plot(yield ~ dose, pch=19, cex=1.25, col="darkblue",
     ylim=yrangeY,  data=seefeldt,
     main="Yield versus Dose")
par(new=T)
plot(xx, yy1, type="l", lwd=3, xlab="", ylab="",
```

```
      ylim=yrangeY, xlim=xrangeD)
  plot(logyield ~ dose, pch=19, cex=1.25, col="darkblue",
      ylim=yrangeLY, data=seefeldt,
      main="Log-Yield versus Dose")
  par(new=T)
  plot(xx, yy2, type="l", lwd=3, xlab="", ylab="",
      ylim=yrangeLY, xlim=xrangeD)
  par(mfrow = c(1,1))

# Figure 7 SE1 Expectation Surface for Theta and Gamma
# Left plot - for Theta
  par(mfrow=c(1, 2))
  eta1   <- seq(0, 1, 0.01)
  eta2   <- eta1^8
  xrange <- range(-0.05, eta1)
  yrange <- range(eta1)
  SE1eta <- function(x, th) exp(-th*x)
  thpts  <- c(20, 3, 1.0, 0.5013037, 0.4, 0.3333, 0.25, 0)
  x1 <- SE1eta(0.50, thpts)
  y1 <- SE1eta(4.0, thpts)
  plot(eta1, eta2, type="l", ylim=yrange, xlim=xrange, lwd=3,
      xlab=expression(eta[1] == eta*(paste("x = 0.5; ", theta))),
      ylab=expression(eta[2] == eta*(paste("x = 4.0; ", theta))) )
  par(new=T)
  plot(x1, y1, xlim=xrange, ylim=yrange, ylab="", xlab="",
      pch=16, cex=2, col="blue")
  par(new=T)
  plot(0.93, 0.025, ylim=yrange, xlim=xrange, ylab="", cex=2,
      xlab="", pch=19)
  text(0.93,   0.07, "y (data)")
  text(0.02,   0.05, expression(paste(theta," = ", infinity)), cex=1.25)
  text(0.2231, 0.05,   expression(paste(theta," = ", 3)),      cex=1.25)
  text(0.58,   0.07,   expression(paste(theta," = ", 1)),      cex=1.25)
  text(0.60,   0.14,   expression(paste(theta," = ", 0.5013)), cex=1.25)
  text(0.6787, 0.2119, expression(paste(theta," = ", 0.40)),   cex=1.25)
  text(0.6900, 0.2736, expression(paste(theta," = ", 0.333)),  cex=1.25)
  text(0.737,  0.3779, expression(paste(theta," = ", 0.25)),   cex=1.25)
  text(0.90,   1,      expression(paste(theta," = ", 0)),      cex=1.25)
  #title("SE1 Expectation Surface (Theta)")
# Right plot: with reparameterization, eta = exp(-x/gamma)
  eta1 <- seq(0, 1, 0.01)
  eta2 <- eta1^8
  xrange <- range(-0.05, eta1)
  yrange <- range(eta1)
  SE1etaNEW <- function(x, gamma) exp(-x/gamma)
  gampts <- c(1/20, 1/3, 1, 1/0.5013037, 1/0.4, 3, 4, 1000)
  x1 <- SE1etaNEW(0.50, gampts)
  y1 <- SE1etaNEW(4.0, gampts)
  plot(eta1, eta2, type="l", ylim=yrange, xlim=xrange, lwd=3,
```

```
      xlab=expression(eta[1] == eta*(paste("x = 0.5; ", gamma))),
      ylab=expression(eta[2] == eta*(paste("x = 4.0; ", gamma))) )
  par(new = T)
  plot(x1, y1, xlim=xrange, ylim=yrange, ylab="", xlab="",
      pch=16, cex=2, col="blue")
  par(new = T)
  plot(0.93, 0.025, ylim=yrange, xlim=xrange, ylab="", cex=2,
      xlab="", pch=19)
  text(0.93,   0.07, "y (data)")
  text(0.00,   0.05, expression(paste(gamma," = ", 0)),       cex=1.25)
  text(0.23,   0.05, expression(paste(gamma," = ", 0.33)),    cex=1.25)
  text(0.54,   0.065, expression(paste(gamma," = ", 1)),      cex=1.25)
  text(0.60,   0.145, expression(paste(gamma," = ", 1.9948)), cex=1.25)
  text(0.6987, 0.2119, expression(paste(gamma," = ", 2.5)),   cex=1.25)
  text(0.75,   0.2836, expression(paste(gamma," = ", 3)),     cex=1.25)
  text(0.78,   0.3879, expression(paste(gamma," = ", 4)),     cex=1.25)
  text(0.90,   1, expression(paste(gamma," = ", infinity)),   cex=1.25)
  par(mfrow = c(1,1))

# Figure 8 SE1 Expectation Surface, Confidence Sphere, etc.
  thE <- 0.5013037
  eta1a <- seq(0, 1, 0.01)
  eta2a <- eta1a^8
  eta1b <- seq(0.4, 1.15, 0.01)
  eta2b <- 1.383855842*eta1b - 0.942414338
  eta1c <- seq(0.3245, 1.5355, length = 10000)
  eta2ctop <- 0.025 + sqrt(0.3666 - (eta1c - 0.930)^2)
  eta2cbot <- 0.025 - sqrt(0.3666 - (eta1c - 0.930)^2)
  XR <- range(-0.10, 1.60)
  YR <- range(-0.60, 1.05)
  plot(eta1a, eta2a, type = "l", ylim = YR, xlim = XR, lwd = 3,
      xlab = expression(eta[1]*paste(" and ")*y[1]),
      ylab = expression(eta[2]*paste(" and ")*y[2]) )
  # tangent line, and then the circle
  par(new = T)
  plot(eta1b, eta2b, type="l", ylim=YR, xlim=XR,
      ylab=" ", xlab=" ", lwd=3, lty=4)
  par(new = T)
  plot(eta1c, eta2ctop, type="l", ylim=YR, xlim=XR,
      ylab=" ", xlab=" ")
  par(new = T)
  plot(eta1c, eta2cbot, type="l", ylim=YR, xlim=XR,
      ylab=" ", xlab=" ")
  # plot the remaining gap
  xxx <- rep(eta1c[10000], 2)
  yyy <- c(eta2ctop[9999], eta2cbot[9999])
  par(new = T)
  plot(xxx, yyy, type="l", ylim=YR, xlim=XR,
      ylab=" ", xlab=" ")
```

```
par(new = T)
# center point
plot(0.77829, 0.13463, ylim=YR, xlim=XR, ylab=" ", xlab=" ",
     cex=1.5, col="darkblue", pch=16)
par(new = T)
# Aside to find coordinates of points C and D on tangent line
fn3 <- function(etaa1) {
  etaa2 <- -0.9424198 + 1.383863*etaa1
  (0.93 - etaa1)^2 + (0.025 - etaa2)^2 - 0.366880394
}
etaa1H <- uniroot(fn3, c(0, 0.778))$root
etaa2H <- -0.9424198 + 1.383863*etaa1H
cbind(etaa1H, etaa2H)
etaa1L <- uniroot(fn3, c(0.778, 2))$root
etaa2L <- -0.9424198 + 1.383863*etaa1L
cbind(etaa1L, etaa2L)
# End of this aside
# Aside to find coordinates of points A and B
# on expectation surface
fn4 <- function(eta1) {
  eta2 <- eta1^8
  (0.93 - eta1)^2 + (0.025 - eta2)^2 - 0.366880394
}
eta1H <- uniroot(fn4, c(0, 0.778))$root
eta2H <- eta1H^8
cbind(eta1H, eta2H)
eta1L <- uniroot(fn4, c(0.778, 2))$root
eta2L <- eta1L^8
cbind(eta1L, eta2L)
# End of this aside
# tangent line points
plot(0.44089, -0.33228, ylim=YR, xlim=XR, ylab=" ", xlab=" ",
     cex=1.5, col="darkblue", pch=16)
par(new = T)
plot(1.11567, 0.601511, ylim=YR, xlim=XR, ylab=" ", xlab=" ",
     cex=1.5, col="darkblue", pch=16)
par(new = T)
# expectation surface points
plot(0.3247, 0.000124,  ylim=YR, xlim=XR, ylab=" ", xlab=" ",
     cex=1.5, col="darkblue", pch=16)
par(new = T)
plot(0.9440, 0.6305,    ylim=YR, xlim=XR, ylab=" ", xlab= " ",
     cex=1.5, col="darkblue", pch=16)
par(new = T)
plot(0.93, 0.025, ylim = YR, xlim = XR, ylab = " ", xlab = " ",
     cex = 1.5, col = "darkblue", pch = 19)
text(1,     0.025, "Y")
text(0.73,  0.19,  "E")
text(0.28,  0.08,  "B")
```

```
  text(0.91,  0.72,  "A")
  text(0.38, -0.32,  "D")
  text(1.11,  0.69,  "C")
  # Visualize tangent line spacing as a function of theta
  aaeta1 <- (1 + 0.5*thE)*exp(-thE*0.5)
  bbeta1 <- -0.5*exp(-thE*0.5)
  eta1point <- function(theta) aaeta1 + bbeta1*theta
  aaeta2 <- (1 + 4*thE)*exp(-thE*4)
  bbeta2 <- -4*exp(-thE*4)
  eta2point <- function(theta) aaeta2 + bbeta2*theta
  # corresponding to 80% WCI (see above)
  cbind(eta1point(-0.3657193), eta2point(-0.3657193)) # (1.1157,0.6015)
  cbind(eta1point( 1.3683267), eta2point( 1.3683267)) # (0.4409,-0.3323)
  (xxx <- c(eta1point(-0.25), eta1point(0),    eta1point(0.25),
            eta1point(0.75),  eta1point(1.00), eta1point(1.25)))
  (yyy <- c(eta2point(-0.25), eta2point(0),    eta2point(0.25),
            eta2point(0.75),  eta2point(1.00), eta2point(1.25)))
  par(new = T)
  plot(xxx, yyy, ylim=YR, xlim=XR, ylab=" ", xlab=" ",
       cex=1.3, col="darkred", pch=15)

# Figure 9 Fieller Creasy Ratio of Two Means
  par(mfrow = c(1, 2))
  y    <- c(3, 4, 5, 6, 6, 7, 8, 8, 9, 10, 10)
  trtA <- c(rep(1, 3), rep(0, 8))
  trtB <- c(rep(0, 3), rep(1, 8))
  FC   <- data.frame(y, trtA, trtB)
  FCmodel <- function(d1, d2, th1, th2) th1*d1 + th1*th2*d2
  fit <- nls(y ~ FCmodel(trtA, trtB, th1, th2), data=FC,
             start = list(th1=1, th2=1))
  summary(fit)
  (LRCI <- confint(fit)[2, ])
  pe   <- coef(fit)[2]
  se   <- sqrt(vcov(fit)[2,2])
  (WCI <- pe + c(-1, 1)*qt(0.975, 9)*se)
  th1E <- coef(fit)[1]
  th2E <- coef(fit)[2]
  # Finding contours
  sseF <- function(th1,th2) {
    ( 3 - th1)^2 +      ( 4 - th1)^2 +      ( 5 - th1)^2 +
    ( 6 - th1*th2)^2 + ( 6 - th1*th2)^2 + ( 7 - th1*th2)^2 +
    ( 8 - th1*th2)^2 + ( 8 - th1*th2)^2 + ( 9 - th1*th2)^2 +
    (10 - th1*th2)^2 + (10 - th1*th2)^2
  }
  WsseF <- function(th1, th2) {
   (3 + 8*th2E^2)*(th1 - th1E)^2 +
    (16*th1E*th2E)*(th1 - th1E)*(th2 - th2E) +
    (8*th1E^2)*(th2 - th2E)^2 +
    20
```

```
  }
  th1 <- seq(1, 7, length = 1000)
  th2 <- seq(0, 6, length = 1000)
  za <- outer(th1, th2, sseF)
  zb <- outer(th1, th2, WsseF)
# Left plot
  contour(th1, th2, za, levels=c(38.91775), lwd=3,
          xlab=expression(theta[1]*paste(": Group A Mean")),
          ylab=expression(theta[2]*paste(": Ratio of Means")))
  points(th1E, th2E, cex=1.5, pch=19, col="blue")
  par(new = T)
  contour(th1, th2, zb, levels=c(38.91775), lty=2, lwd=3,
          col="darkgreen")
  legend("topright",
         legend = c("95% Likelihood CR","95% Wald CR"),
         lwd=3, col=c("black", "darkgreen"), lty=c(1, 2))
# Right plot
  plciL95 <- 1.300386;  plciR95 <- 3.941499
  wciL95  <- 0.9819219; wciR95  <- 3.018071
  plciL99 <- 1.112213;  plciR99 <- 6.714884
  wciL99  <- 0.5374069; wciR99  <- 3.462591
  # Shifted profiled SSE and Wald approx for theta2 for plotting
  th2LR <- seq(1.1, 7, length = 1000)
  profileSSLR.FC <-  3*8*th1E*th1E*((th2LR - th2E)^2)/(3 + 8*th2LR*th2LR)
  th2W <- seq(0.5, 3.5, length = 1000)
  profileSSW.FC <- (3*8*th1E*th1E/(3 + 8*th2E*th2E))*(th2W - th2E)^2
  xrange <- range(0, th2LR, th2W)
  yrange <- range(profileSSLR.FC, profileSSW.FC, 27)
  c95 <- (20/9)*qt(0.975, 9)^2
  c99 <- (20/9)*qt(0.995, 9)^2
  plot(th2LR, profileSSLR.FC, type = "l", lwd = 3,
       xlim = xrange, ylim = yrange,
       xlab = expression(theta[2]*paste(": Ratio of Means")),
       ylab = "Shifted PSSE")
  par(new = T)
  plot(th2W, profileSSW.FC, type = "l", lwd = 3,
       xlim = xrange, ylim = yrange,
       lty = 2, xlab = "", ylab = "")
  points(th2E,      0, pch=19, cex=2, col= "darkgreen")
  points(plciL95, c95, pch=19, cex=2, col= "darkblue")
  points(plciR95, c95, pch=19, cex=2, col= "darkblue")
  points(wciL95,  c95, pch=19, cex=2, col= "purple")
  points(wciR95,  c95, pch=19, cex=2, col= "purple")
  points(plciL99, c99, pch=19, cex=2, col= "darkblue")
  points(plciR99, c99, pch=19, cex=2, col= "darkblue")
  points(wciL99,  c99, pch=19, cex=2, col= "purple")
  points(wciR99,  c99, pch=19, cex=2, col= "purple")
  abline(h = c99, col = "black", lty = 3)
  x95 <- seq(0.7, 4.2, length = 1000)
```

```
cut95 <- rep(c95, 1000)
par(new = T)
plot(x95, cut95, type="l", lty=3, xlim=xrange, ylim=yrange,
     xlab="", ylab="")
legend("bottomright", legend=c("Profiled SSE", "Wald Approx."),
       lty=c(1, 2), lwd=3)
legend(4.3, c95 + 1.7, legend = c("95% cut line"))
legend(3.75, 27.5, legend = c("99% cut line"))
text( wciL99  - 0.45, c99 + 1.2, expression(C[99]))
text( wciR99  - 0.45, c99 + 1.2, expression(D[99]))
text( plciL99 + 0.45, c99 + 1.2, expression(A[99]))
text( plciR99 + 0.15, c99 + 1.7, expression(B[99]))
text( wciL95  - 0.5,  c95 + 1.2, expression(C[95]))
text( wciR95  - 0.45, c95 + 1.2, expression(D[95]))
text( plciL95 + 0.45, c95 + 1.2, expression(A[95]))
text( plciR95 - 0.45, c95 + 1.2, expression(B[95]))
```
